# Integrated *in silico*–*in vitro* and pharmacokinetic profiling of *Thymus vulgaris*-derived metabolites targeting multidrug resistance pathways in extensively drug-resistant *Acinetobacter baumannii* (muks92)

**DOI:** 10.3389/fmicb.2025.1680686

**Published:** 2025-12-17

**Authors:** Ali Hazim Abdulkareem, Mohammed Mukhles Ahmed, Safaa Abed Latef Al-Meani, Elham Hazeim Abdulkareem

**Affiliations:** 1 Department of Biotechnology, College of Science, University of Anbar, Ramadi, Iraq; 2 Department of Oral and Maxillofacial Surgery, College of Dentistry, University of Anbar, Ramadi, Iraq

**Keywords:** *Thymus vulgaris*, *Acinetobacter baumannii*, extensively drug-resistant (XDR), β-lactamase, GC–MS, molecular docking, biofilm inhibition

## Abstract

**Background:**

*Acinetobacter baumannii* is a major nosocomial pathogen with extensive drug resistance (XDR) driven by β-lactamases, efflux systems, and biofilm formation. Plant-derived metabolites are promising multi-target modulators of these pathways.

**Methods:**

Ten XDR *A. baumannii* isolates with strong biofilm and β-lactamase activity were screened; the most resistant (strain muks92) underwent whole-genome sequencing and *in-silico* analyses. Essential oil from *Thymus vulgaris* was profiled by GC–MS, identifying o-cymene (32.95%) and gamma-terpinene (16.85%) as dominant constituents. Molecular docking (AutoDock Vina) targeted class D β-lactamase (6T1H), a biofilm-associated regulator (5HM6), and PBP1a/efflux-associated protein (8YR0), with post-docking visualization and AMBER-relaxed stability checks. Antibiofilm activity was quantified by crystal-violet microtiter assay, and ESBL activity by nitrocefin hydrolysis. Multilocus sequence typing (MLST), resistance/virulence gene mining, and mobile genetic element mapping were performed on the genome. Pharmacokinetic/toxicity properties for key metabolites were predicted using ADMET-AI.

**Results:**

GC–MS established a cymene/terpinene-rich chemotype. Docking showed favorable hydrophobic/*π*–alkyl encapsulation of o-cymene and gamma-terpinene within catalytic or transport pockets of 6T1H, 5HM6, and 8YR0, consistent with steric gating of substrate access. *T. vulgaris* significantly reduced biofilm biomass versus control (*p* = 0.0002), and lowered ESBL activity (*p* = 0.0017). The muks92 genome (3.98 Mb; ST1104) carried a complex resistome including blaOXA-90, blaOXA-72, blaADC-25, blaCARB-14, armA, aac(6′)-Ian, aadA5, sul1/sul2, mph(E)/msr(E), alongside virulence loci (bap, bfmRS, ompA, pgaABCD, csuA/B–E) and siderophore systems (bas/bau/bar), with multiple insertion sequences and an IncA/C2 replicon. ADMET predictions supported good oral absorption and low acute toxicity; gamma-terpinene showed broader tissue distribution (predicted VDss ≈ 7.24 L/kg) and a longer half-life (~4.6 h) than o-cymene.

**Conclusion:**

*T. vulgaris* metabolites, particularly gamma-terpinene, exhibit multi-target inhibitory potential against resistance and virulence pathways in XDR *A. baumannii* (muks92), aligning *in-silico* interactions with measurable antibiofilm and ESBL attenuation *in vitro*. Given the predictive nature of docking/ADMET outputs, targeted enzyme kinetics, standardized MIC/MBC testing, cytotoxicity assays, and in-vivo validation are warranted before therapeutic translation.

## 1 Introduction

*Acinetobacter baumannii* has emerged as one of the most critical nosocomial pathogens globally, particularly due to its remarkable ability to acquire multidrug resistance (MDR) and survive in harsh hospital environments (Peleg et al., 2008). As of May 17, 2024, the World Health Organization (WHO) updated its Bacterial Priority Pathogens List (BPPL), reaffirming the classification of carbapenem-resistant *Acinetobacter baumannii* (CRAB) as a “Critical Priority 1” pathogen. Such a title highlights the urgent necessity of new treatment measures to counter the infections of this multidrug-resistant bacterium (Sati et al., 2025). Resistance among this species is frequently multifactorial with extended-spectrum 8-lactamase (ESBL) production, biofilm formation, and active efflux pump mechanisms, all of which are interrelated in terms of its persistence and treatment failure in the clinical setting (Antunes et al., 2014; Vuotto et al., 2014; Coyne et al., 2011).

*Acinetobacter baumannii* has been identified to have both inbuilt and acquired antibiotic resistance profile. These comprise enzyme-mediated breakdown of antibiotics, changes in the binding sites of targets, alternative outer membrane permeability and overexpression of efflux pump proteins. The beta-lactam-resistant bacteria are a global health issue as they cause a high percentage of hospital-acquired infections (Lowings et al., 2015). Various beta-lactam resistance systems have been detected in *A. baumannii*. Of them, one of the most common determinants that confer resistance to a variety of *β*-lactam antibiotics is oxacillinases (OXAs) including *oxa-23-like* and *oxa-51-like* genes (Khurshid et al., 2017). This means that the growing resistance of strains to *β*-lactam-based positions the efficacy of antibiotic-based treatment as a limiting factor in most instances, with colistin being the final option of treatment (Ababneh et al., 2021).

The growing number of extensively drug-resistant (XDR) *A. baumannii*, non-susceptible to one agent in all but two or fewer categories of antimicrobials (Magiorakos et al., 2012), underlines the urgency of alternative or complementary therapies. The trends of interest in phytochemicals obtained through medicinal plants as possible antibacterial agents is associated with multitarget actions and reduced propensity to cause resistance (Khameneh et al., 2019). These include *Thymus vulgaris* (common thyme), a commonly used culinary and medically significant herb, which has a wide range of bioactive compounds such as thymol and carvacrol which have a broad-spectrum antimicrobial activity (Kosker et al., 2016; Soković et al., 2010).

The essential oils of *T. vulgaris* have been shown in previous research to inhibit Gram-positive and Gram-negative bacteria by disrupting membrane integrity, disrupting the quorum sensing and regulating expression of the efflux pump (Bajpai et al., 2012; Cardinale et al., 2006; Nazzaro et al., 2013). Nevertheless, it is still not fully assessed in terms of its impact on clinically relevant resistance mechanisms, especially in XDR *A. baumannii*.

The proposed research will be able to fill this gap by offering a twofold approach: *in vitro* evaluation of anti-bacterial, anti-biofilm and efflux pump-inhibitory properties of *T. vulgaris* essential oils and in silico molecular docking of its major phytochemicals with those of major proteins (associated with resistance). A well-characterized clinical isolate, *A. baumannii* strain muks92, displaying ESBL production, biofilm formation, and XDR phenotype, was selected for this purpose. The integration of computational and experimental analyses in this study offers novel insight into the mechanistic potential of thyme-derived compounds as promising candidates in combating multidrug-resistant *A. baumannii*.

## 2 Materials and methods

**Figure 1:**
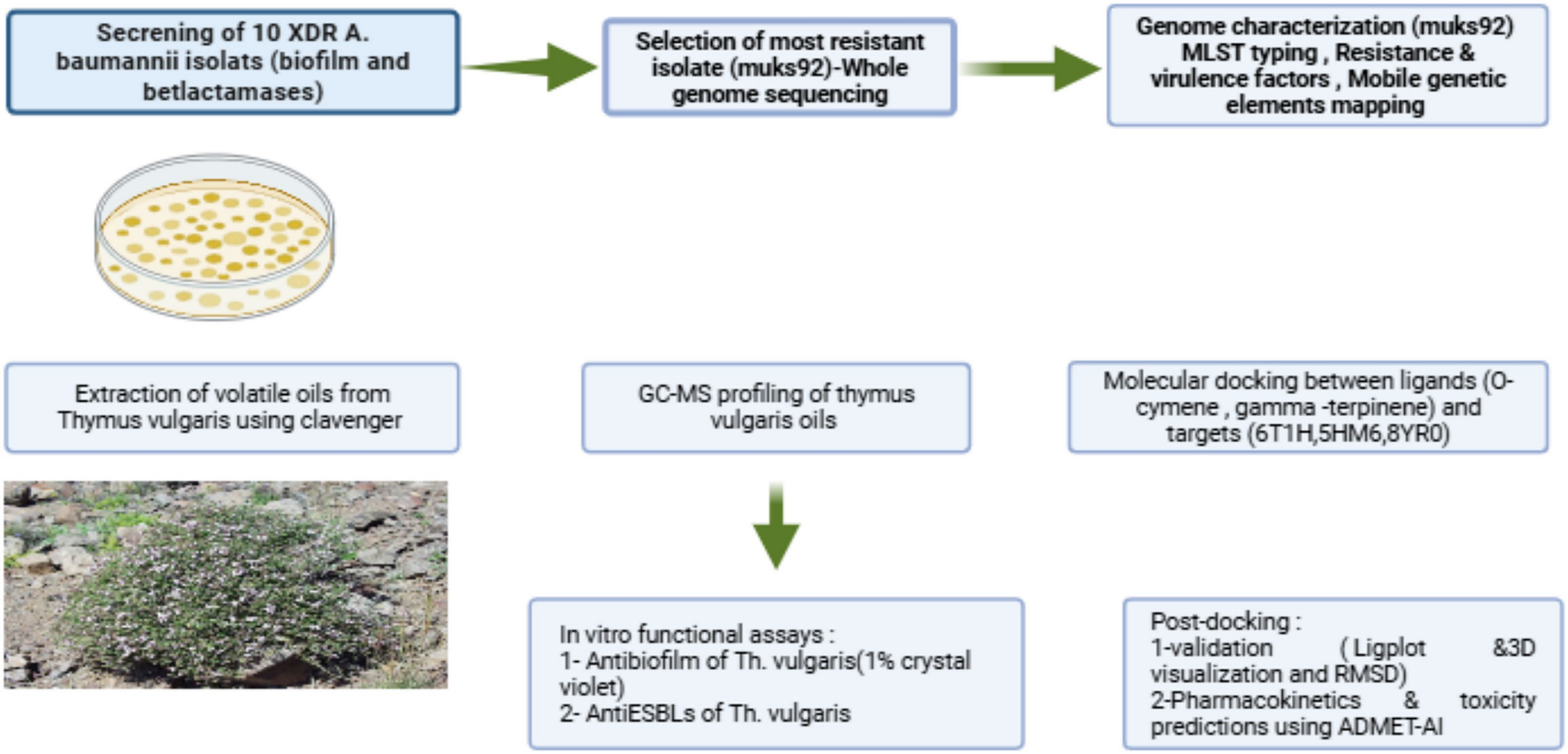
Integrated workflow of experimental and computational approaches. The schematic outlines the study design beginning with the screening of ten extensively drug-resistant *Acinetobacter baumannii* isolates for biofilm and β-lactamase activity, leading to the selection of strain muks92 for whole-genome sequencing and genomic characterization. Essential oil of *Thymus vulgaris* was extracted and profiled by GC–MS, identifying o-cymene and gamma-terpinene as major metabolites. These compounds were subjected to molecular docking against β-lactamase (6T1H), biofilm regulator (5HM6), and PBP1a/efflux protein (8YR0), followed by post-docking validation and stability checks. The findings were complemented by functional assays (antibiofilm activity), and prediction of *in silico* ADMET.

Figure 1 illustrates the integrated experimental and computational pipeline applied in this work. Initially, ten extensively drug-resistant (*A. baumannii*) isolates were screened for biofilm formation and *β*-lactamase activity, leading to the selection of the most resistant strain (muks92) for whole-genome sequencing. Genomic characterization included multilocus sequence typing (MLST), detection of resistance and virulence determinants, and mapping of mobile genetic elements. In parallel, volatile oils were extracted from *Thymus vulgaris* using a Clevenger apparatus, followed by GC–MS profiling, which identified o-cymene (32.95%) and gamma-terpinene (16.85%) as the dominant constituents. These metabolites were subjected to molecular docking (AutoDock Vina) against class D *β*-lactamase (6T1H), a biofilm-associated regulator (5HM6), and PBP1a/efflux-associated protein (8YR0). Post-docking analysis involved LigPlot diagrams, 3D visualization, and stability validation through AMBER relaxation. Functional assays quantified the antibiofilm potential of *T. vulgaris* essential oils using crystal violet staining and evaluated anti-ESBL activity via nitrocefin hydrolysis. Finally, pharmacokinetic and toxicity properties of the major metabolites were predicted using ADMET-AI.

### 2.1 Bacterial isolation, culture, and DNA extraction

The extensively drug-resistant (XDR) *Acinetobacter baumannii* strain muks92 was isolated from a blood sample collected from a female patient suffering from bacteremia following exposure to an explosive device in Al-Anbar Province, Iraq. The sample was obtained at Ramadi Teaching Hospital. Isolation was performed by culturing the sample on MacConkey agar and blood agar (Oxoid, UK) and incubating at an aerobic temperature of 37 o C and 44 o C of 24 hours. The morphology of *Acinetobacter* spp. was observed because the organism formed lactose-negative colonies on MacConkey agar and non-hemolytic grayish colonies on blood agar. One colony was further sub-cultured on nutrient agar.

The VITEK 2 Compact system (bioMerieux) was used to confirm the identification. The VITEK 2 system that evaluated sensitivity to an extensive collection of antibiotics was used to perform antimicrobial susceptibility testing. The guidelines of Clinical and Laboratory Standards Institute (CLSI) were used to interpret.

The isolate was sensitive to all the antibiotics except tigecycline. The clinical severity of the case is emphasized by the fact that the patient died of the infection despite the treatment. The manufacturer used the Geneaid DNA extraction kit (Geneaid, Taiwan) to extract genomic DNA based on the instructions provided by the manufacturer. The quality and concentration of DNA were determined by Qubit Fluorometer and NanoDrop spectrophotometer (both Thermo Fisher Scientific, USA). Whole-genome sequencing of the DNA sample was done at BGI Genomics (Poland).

The institutional review board (IRB) of the Ramadi teaching hospital and the college of science, university of Anbar approved this study (Approval No.: ANB2024-003). Informed consent was given as a waived time and again because of retrospective and anonymized sample.

### 2.2 Whole genome sequencing and assembly

Whole-genome sequencing of *A. baumannii* muks92 was performed using the DNBSEQ platform. To prepare the paired-end library, the genomic DNA was broken down into about 350 bp fragments (150 bp 2). The quality control was done by SOAPnuke (v1.5.6), which produced 1,288 Mb of clean reads and a final coverage of the genome of 323X. Adapters and low quality reads and duplicates were filtered out. *De novo* assembly was performed using SPAdes (v3.9.0). Gene prediction was carried out using Glimmer3, while non-coding RNAs were annotated with tRNAscan-SE, RNAmmer, and Infernal/Rfam. Tandem repeats were identified using Tandem Repeats Finder (TRF).

### 2.3 Genome annotation and functional analysis

Functional annotation of protein-coding genes was performed using multiple databases: the Virulence Factor Database (VFDB) for virulence-associated genes, the Antibiotic Resistance Genes Database (ARDB) for resistance determinants, and the Carbohydrate-Active enZymes (CAZy) database for metabolic enzymes. Additional annotations included Swiss-Prot, COG, and KEGG databases. Genome quality metrics were assessed using k-mer analysis to estimate genome size and confirm sequencing accuracy. GC-depth correlation analysis was used to evaluate GC bias. The final genome assembly statistics are summarized in Table 1.

**Table 1:** Summary of genome sequencing and annotation for XDR *Acinetobacter baumannii* strain muks92.

Category	Details
Scaffolds	108
Genome size	3,981,734 bp
N50	94,472 bp
GC content	39.00%
Protein-coding genes	3,717 (81.16% annotated)
tRNAs	62 (tRNAscan-SE)
rRNAs	1 × 16S, 1 × 23S, 2 × 5S (RNAmmer)
sRNAs	99 (Infernal/Rfam)
Tandem repeats	100 (TRF); 63 minisatellites, 11 microsatellites
Virulence factors	272 (7.31%) – VFDB
Antibiotic resistance	25 (0.67%) – ARDB
CAZymes	119 (3.2%) – CAZy
Swiss-prot matches	1,578 (42.45%)
COG matches	2,791 (75.08%)
KEGG matches	2,244 (60.37%)
Estimated genome size	~4.21 Mb (k-mer analysis)
Sequencing depth	47.5×
GC bias	None detected

### 2.4 Multilocus sequence typing (MLST) and phylogenetic identification

Multilocus sequence typing (MLST) of the isolate was conducted *in silico* using the whole-genome sequence data. Both the Pasteur and Oxford MLST schemes were applied through the MLST 2.0 tool (version 2.0.1) available on the Center for Genomic Epidemiology (CGE) platform.^1^ For phylogenomic analysis based on whole-genome data, the assembled genome was submitted to the Type Strain Genome Server (TYGS).^2^ Taxonomic identification of the nearest type strains and genome-wide comparisons were performed using the Genome BLAST Distance Phylogeny (GBDP) method, employing the ‘coverage’ algorithm and the distance formula d5.

### 2.5 Genome analysis

Genome annotation was carried out automatically using the Prokaryotic Genome Annotation Pipeline (PGAP) provided by the National Center for Biotechnology Information (NCBI), employing the GeneMarkS-2 + algorithm (version 6.1) with the best-placed reference protein set. Circular representations of both the chromosome and plasmids were generated using the CGView visualization tool.^3^ Predicted protein-coding sequences (CDSs) were functionally annotated based on the Clusters of Orthologous Groups (COG) database.

Antibiotic resistance genes (ARGs) were identified using ResFinder^4^ and the Comprehensive Antibiotic Resistance Database (CARD),^5^ with genome data submitted and analyzed on July 4, 2022. Insertion sequences (IS elements) were detected using the IS Finder platform.^6^ Prediction of virulence-associated genes was performed using BLASTp against the Virulence Factor Database (VFDB).^7^

To identify prophage regions, the genome was screened using the PHASTER tool.^8^ Genomic islands (GIs) were predicted through IslandViewer4.^9^ Additionally, clustered regularly interspaced short palindromic repeats (CRISPRs) were detected using CRISPRCasFinder.^10^

### 2.6 Molecular docking

#### 2.6.1 Ligand preparation

The chemical structures of o-cymene (C₁₀H₁₄; PubChem CID: 7463) and gamma-terpinene (C₁₀H₁₆; PubChem CID: 7461) were retrieved in SMILES format from the PubChem database. These structures were converted into 3D conformations using Open Babel, followed by energy minimization with the MMFF94 force field to obtain the most stable geometry.

#### 2.6.2 Protein structure retrieval and preparation

Three essential proteins of *Acinetobacter baumannii* were selected as molecular targets for docking analysis: class D *β*-lactamase (PDB ID: 6T1H), biofilm formation (PDB ID: 5HM6), and penicillin-binding protein 1a (PBP1a; PDB ID: 8YR0). The corresponding crystal structures were retrieved from the Protein Data Bank (PDB) and prepared using UCSF Chimera. Prior to docking, all water molecules, ions, and non-standard residues were removed. Polar hydrogen atoms were added, and Gasteiger partial charges were assigned. The processed protein structures were then saved in PDBQT format for subsequent molecular docking experiments.

#### 2.6.3 Docking protocol

Table 2 shows three key proteins of *Acinetobacter baumannii* were selected as molecular targets for docking studies: class D *β*-lactamase (PDB ID: 6T1H), biofilm formation (PDB ID: 5HM6), and penicillin-binding protein 1a (PBP1a; PDB ID: 8YR0). The crystal structures of these proteins were retrieved from the Protein Data Bank (PDB) and prepared using UCSF Chimera. During preprocessing, all water molecules, ions, and non-standard residues were removed. Polar hydrogen atoms were added, and Gasteiger charges were assigned. The structures were then converted to PDBQT format for docking.

**Table 2:** Docking parameters used for molecular docking analysis of *Acinetobacter baumannii* targets.

Target protein	PDB ID	Grid center coordinates (x, y, z)	Grid box size (Å)	Exhaustiveness	Number of poses generated
Class D β-lactamase	6T1H	(22, −21, −8)	20 × 20 × 20	16	20
Biofilm-controlling response regulator	5HM6	(20, 46, −16)	20 × 20 × 20	16	20
Penicillin-binding protein 1a (PBP1a)	8YR0	(218, 223, 208)	20 × 20 × 20	16	20

Molecular docking was performed using AutoDock Vina (version 1.2.0). The active sites of each protein were defined by grid boxes centered on the catalytic or ligand-binding domains, guided by prior literature and visual inspection. The grid parameters were as follows: for beta lactamases (6T1H), center = (22, −21, −8); for biofilm formation (5HM6), center = (20, 46, −16); and for PBP1a (8YR0), center = (218, 223, 208), each with a box size of 20 × 20 × 20 Å. An exhaustiveness value of 16 was used to ensure sufficient conformational sampling. Each ligand was docked independently to the three protein targets, and AutoDock Vina generated 20 binding poses per ligand, which were ranked according to their predicted binding affinities (kcal/mol).

#### 2.6.4 Post-docking analysis

The best-ranked poses were selected for visualization and interaction analysis. Three-dimensional (3D) interaction plots were rendered using PyMOL, while two-dimensional (2D) interaction diagrams were generated using BIOVIA Discovery Studio Visualizer. Key binding residues, interaction types (hydrophobic, van der Waals, alkyl, *π*-alkyl), and spatial orientation were examined in detail.

#### 2.6.5 Validation and references

Docking protocols were validated by cross-referencing known binding residues from literature.

#### 2.6.6 RMSD analysis after AMBER relaxation

Following energy minimization and equilibration under the AMBER relaxation protocol, the structural stability of each protein–ligand complex was evaluated using root mean square deviation (RMSD) analysis. The equilibrated structure obtained after AMBER relaxation was taken as the reference. Trajectories from production runs were aligned to this relaxed structure using backbone atoms (Cα, C, N), and RMSD values were calculated with CPPTRAJ to monitor deviations over time.

### 2.7 The toxicity and pharmacokinetic of ligands

These pharmacokinetic and toxicity profiles of the compounds were determined by the ADMET-AI online prediction system.^11^ The chemical structures were input in SMILES format, allowing batch submission of multiple molecules. The platform integrates graph neural network-based modeling (Chemprop) with approximately 200 physicochemical descriptors derived from RDKit to estimate a wide range of ADMET parameters. The prediction model was developed and trained on validated datasets from the Therapeutics Data Commons, covering both regression tasks (e.g., solubility, clearance, volume of distribution) and classification endpoints (e.g., Ames toxicity, hERG inhibition, CYP450 enzyme inhibition). Each molecule was evaluated individually, and the predicted results were downloaded in tabulated form for analysis. The platform offers efficient processing and supports screening of up to 1,000 compounds per batch.

### 2.8 Collection of *Thymus vulgaris*

The leaves of this plant were collected from the western desert of Al-Anbar, near the city of Rutba. The species was taxonomically identified by a plant classification specialist. The collected leaves were then air-dried and ground into fine powder.

### 2.9 Extraction of active using clevenger-type hydrodistillation apparatus

*Thymus vulgaris* leaves were processed for essential oil extraction using a Clevenger-type hydrodistillation apparatus under controlled conditions, following the protocol reported by Elyemni et al. (2019). A sample of 100 g of *Thymus vulgaris* leaves material was combined with 800 mL of distilled water and subjected to distillation at 40 °C for a duration of 3 h. the distillate was subsequently collected, and the essential oil fraction was separated and dried over anhydrous sodium sulfate to eliminate residual moisture. Amber glass vials were used to store the obtained oil at 4 o C to prevent oxidative degradation and maintain volatile constituents until further analysis.

### 2.10 Analysis of active compounds derived—*Thymus vulgaris* by gas chromatography–mass spectrometry (GC–MS)

The chemical constituents of the *Thymus vulgaris* were profiled using a GC–MS instrument (Agilent 7820A, Santa Clara, CA, USA) fitted with an HP-5 ms Ultra Inert capillary column (30 μm × 250 μm × 0.25 μm). For each run, 1 μL of the sample was injected in split mode, employing helium (99.99% purity) as the carrier gas at a constant pressure of 11.933 psi. The injector temperature was maintained at 250 °C, and the transfer line (auxiliary heater) was kept at 310 °C. Mass spectra were acquired over the m/z range of 50–500. The oven temperature program was initiated at 60 °C and held for 1 min, followed by a linear increase to 180 °C at a rate of 7 °C/min, and subsequently raised to 280 °C at the same ramping rate, resulting in a total chromatographic run time of nearly 33 min (Muhmood et al., 2025).

### 2.11 Estimation of minimum inhibitory concentration for *Thymus vulgaris* using resazurin method

The minimum inhibitory concentration (MIC) of *Thymus vulgaris* was assessed using the broth microdilution technique in accordance with the Clinical and Laboratory Standards Institute (CLSI) guidelines. A series of two-fold dilutions of ellagic acid, ranging from 4 to 100 μg/mL, were prepared in tryptic soy broth. Each dilution was inoculated with a standardized bacterial suspension adjusted to 0.5 McFarland standard (approximately 1.5 × 10^8^ cells/mL) in sterile 96-well U-bottom microtiter plates. The plates were incubated at 37 °C for 18 h. Following incubation, 20 μL of resazurin blue solution was added to each well, and the plates were further incubated for an additional 2 to 4 h. Viable bacteria reduce the blue, non-fluorescent resazurin to pink, fluorescent resorufin, which may be further reduced to colorless hydroresorufin, serving as an indicator of bacterial metabolic activity (Gupta et al., 2018; Sampaio et al., 2021). cefotaxime (CLSI-recommended concentrations) served as positive controls to confirm growth inhibition. Wells with sterile broth and resazurin only acted as negative controls to exclude contamination or background change. Growth controls contained bacterial inoculum without extract or antibiotic to verify normal viability and resazurin reduction.

### 2.12 Treatment of ESBLs with *Thymus vulgaris*

Based on Epp et al. (2021) and Abdulkareem et al. (2023). The enzymatic activity of *β*-lactamase in *Acinetobacter baumannii strain: muks92* was evaluated by monitoring the hydrolysis of nitrocefin, a chromogenic *β*-lactam substrate. The reaction mixture consisted of nitrocefin, bovine serum albumin (BSA), glycerol, and bacterial cell lysates, all prepared in phosphate buffer. The assay was conducted at temperature 37 °C, and the reaction progress was monitored by measuring the decrease in absorbance at 390 nm at 10-min intervals. Enzymatic activity was expressed as the rate of nitrocefin hydrolysis, calculated in micromoles per minute per milligram of protein. *K. pneumoniae ATCC 25922* was used as a reference control strain to ensure reproducibility of the results. Additionally, the effect of sub-inhibitory concentrations (sub-MICs) of *Thymus vulgaris* on extended-spectrum *β*-lactamase (ESBL)-producing strains was assessed to explore potential inhibitory effects. For validation of the *β*-lactamase assay, positive controls were set by incubating nitrocefin with ESBL-producing *A. baumannii* lysate to establish baseline enzyme activity. Negative controls consisted of nitrocefin in phosphate buffer without enzyme, confirming substrate stability and absence of spontaneous hydrolysis. In addition, a reference inhibition control using nitrocefin with bacterial lysate and clavulanic acid was included to demonstrate the assay’s capacity to detect *β*-lactamase inhibition.

### 2.13 Treatment of biofilm with *Thymus vulgaris*

A modified microtiter plate assay was employed to assess biofilm formation. Each well received 190 μL of fresh brain heart infusion (BHI) broth supplemented with 1% glucose, along with 32 μg of *Thymus vulgaris* essential oils. Subsequently, 10 μL of an overnight culture of *Acinetobacter baumannii* strain muks92 was added. In the control wells, BHI broth was used that did not have the essential oils. The plates were stirreded at 200 rpm over a period of 1 h and incubated at 37 0 C over a period of 18 h. The non-adherent planktonic cells were washed out by placing the wells into sterile water after incubation. The rest of the biofilms were stained using 1 percent crystal violet during a period of 15 min and then other washing procedures were taken. Quantitative evaluation was then done by solubilizing the bound dye with 200 μL of 95% ethanol (Nor Amdan, 2018). The quantitative method of measuring biofilm formation was by measuring optical density (OD) at 630 nm. Results interpretation was pegged on a cut-off OD value (ODc), which is taken as the average absorbance of the negative control wells. Due to this cut-off, isolates were grouped into four categories, namely, non-biofilm producers (OD ≤ ODc), weak biofilm producers (ODc < OD ≤ 2 × ODc), moderate biofilm producers (2 × ODc OD ≤ 4 × ODc), and strong biofilm producers (OD > 4 × ODc) (Abdulkareem et al., 2023). In the biofilm assay, the positive controls would be wells with the *A. baumannii* strain muks92 incubated in the BHI together with glucose and without essential oils, to promote the growth of a strong biofilm. Negative controls contained sterile BHI broth only, confirming absence of background staining. A reference inhibition control was included by treating cultures with a known antibiofilm agent (ciprofloxacin, gentamicin, or dispersin B) to benchmark the inhibitory effect of *T. vulgaris* essential oils.

### 2.14 Ethics approval

The research was approved by the Scientific Research Ethics Committee at the University of Anbar under approval number 647.

### 2.15 Statistical procedure

Statistical analysis was performed using GraphPad Prism (version 8, GraphPad Software, San Diego, CA, USA). All experiments were carried out in triplicate, and data are expressed as mean ± standard error of the mean (SEM). Prior to analysis, the normality of data distribution was assessed using the Shapiro–Wilk test. For comparison between two groups, an unpaired Student’s *t*-test was applied. In cases where data did not follow a normal distribution, the non-parametric Mann–Whitney U test was used. For multiple group comparisons, one-way ANOVA followed by Tukey’s *post hoc* test was employed. Statistical significance was considered at *p* < 0.05, and highly significant differences were indicated at *p* < 0.01. Graphs were generated in GraphPad Prism with error bars representing SEM.

## 3 Results

### 3.1 Antibiotic resistance profile

This isolate was resistant to all tested antibiotics except for tigecycline as XDR ISOLATE.

#### 3.1.1 Gas chromatography–mass spectrometry (GC–MS) profile of *Thymus vulgaris* essential oils

**Figure 2:**
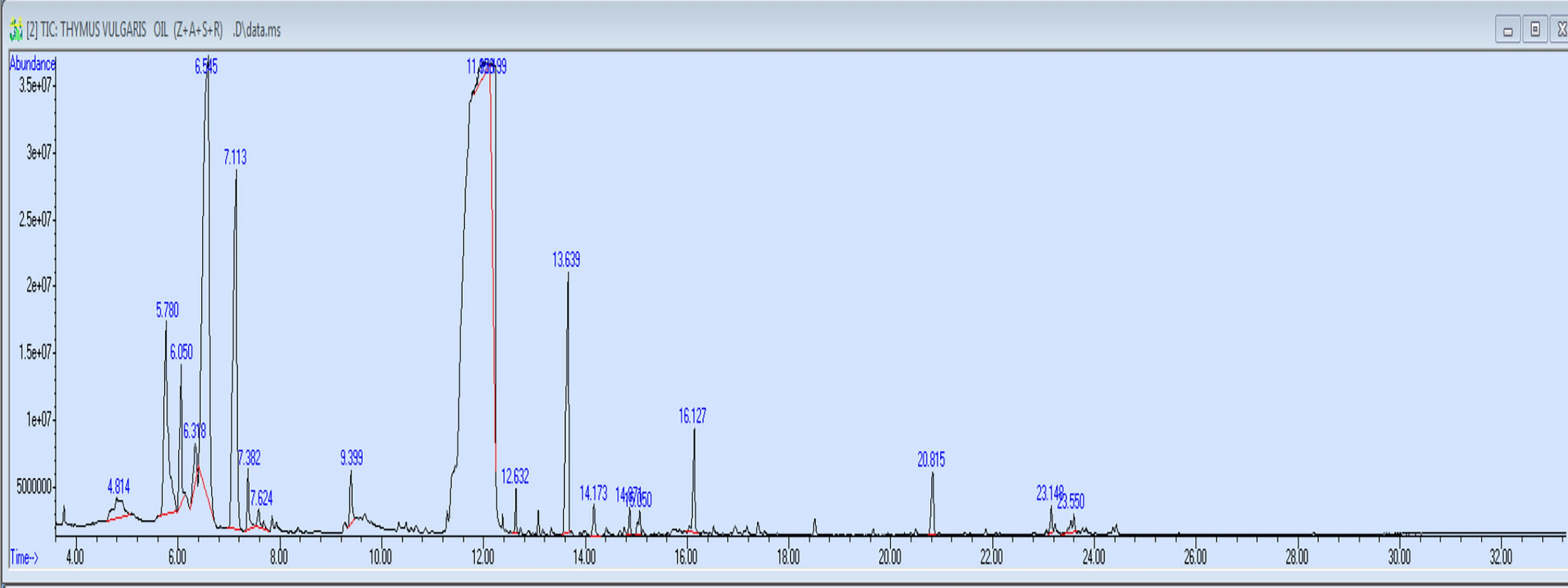
GC–MS analysis of thyme essential oils.

Table 3 and Figure 2 show the chromatographic separation of *Thymus vulgaris* essential oil revealed a complex mixture of monoterpenes, sesquiterpenes, and phenolic derivatives, with distinct retention behaviors and spectral matches.

**Table 3:** Active compounds of thyme essential oil using GC–MS technique.

Peak	RT (min)	Area %	Compound name (Tentative ID)	CAS No.	Kovats index (KI, lit.)	Quality match	Remarks/co-elution notes
1	4.816	2.37	α-Pinene	80–56-8	~939	64	Co-elution with bicyclic terpenes (possible mis-attribution).
2	5.777	9.92	1-Octen-3-ol	3,391-86-4	~979	58	Overlap with 1-Nonen-3-ol, requires confirmation.
3	6.054	3.51	3-Octanol	589–98-0	~982	74	Likely pure peak, well-matched.
4	6.322	1.48	2-Carene / p-Menthadiene isomers	554–61-0	~1,011	93–95	Possible isomeric overlap with 1-methyl-4-isopropyl-cyclohexadiene.
5	6.547	32.95	o-cymene (major)	527–84-4	~1,026	59–80	Strong peak, possible tetramethyl-benzene co-elution.
6	7.109	16.85	gamma-terpinene	99–85-4	~1,059	80–94	Major monoterpene, consistent with thyme oil.
7	7.378	1.47	p-Menth-8-en-1-ol (isomers)	7,299-40-3	~1,145	93–94	Several stereoisomers overlapped, requires verification.
8	7.620	0.63	Terpinolene / related	586–62-9	~1,088	93–96	Minor monoterpene, slight co-elution.
9	9.403	1.32	Terpinen-4-ol	562–74-3	~1,177	89–94	Well-identified, consistent with thyme chemotype.
10	11.956	1.56	2,3,5,6-Tetramethylphenol (trace phenol)	527–35-5	~1,260	59–90	Minor phenolic component.
11	12.199	11.30	Thymol / isomeric tetramethyl phenols	528–75-2	~1,290	87	Likely Thymol (key marker). Possible overlap with carvacrol.
12	12.632	0.72	Eugenol	97–53-0	~1,356	96–98	Confirmed (literature-consistent).
13	13.636	8.18	β-Caryophyllene	87–44-5	~1,418	94–99	Major sesquiterpene, well-identified.
14	14.172	0.81	Humulene (α-Caryophyllene)	6,753-98-6	~1,452	74–98	Correct assignment, minor sesquiterpene.
15	14.873	0.53	β-Bisabolene	495–61-4	~1,505	70–93	Minor sesquiterpene, slight overlap with myrcene.
16	15.046	0.66	Hexahydronaphthalenes (isomers)	483–76-1	~1,525	89–97	Tentative; requires standard comparison.
17	16.128	2.66	Caryophyllene oxide	1,139-30-6	~1,582	87–91	Sesquiterpene oxide, confirmed.
18	20.811	1.75	Artefact / non-volatile (low match)	–	–	10	Likely mis-assignment, exclude from discussion.
19	23.148	0.55	Methyl oleate (ester)	112–62-9	~2,110	99	Long-chain ester, possibly column carry-over.
20	23.546	0.79	Artefact peaks (low match, <20%)	–	–	<20	Likely contaminants or noise, exclude.

The first eluted component, α-pinene (RT 4.816 min, 2.37%), is a bicyclic monoterpene hydrocarbon frequently found in Lamiaceae oils. Its Kovats index (~939) corresponds well with reported values, but spectral matching (64%) suggests possible co-elution with structurally related bicyclic terpenes. Despite its relatively low proportion, α-pinene contributes to the characteristic resinous aroma and is known for anti-inflammatory properties.

At RT 5.777 min, a more abundant fraction (9.92%) was tentatively identified as 1-octen-3-ol (KI ~ 979). This alcohol is a common fungal metabolite but has also been reported in thyme oil. This average quality of the spectral (58%) and coincidence with 1-nonen-3-ol show the necessity to confirm the results with genuine standards further. It adds a mushroom-like smoky flavor and could contribute to the complexity of thyme essential oils.

3-Octanol was eluted at 6.054 min and had a contribution area of 3.51. It has a high match factor (74%), and its Kovats index (~982) indicates credible identification. Being a saturated aliphatic alcohol, it itself has no direct bioactivity, but may be useful in increasing the solubility of other volatile constituents.

The eluting component with a retention time of 6.322 min (1.48 percent) was attributed to 2-carene/p-menthadiene isomers (KI = 1,011). This presence is suggested by high-quality matches (9395%), but structural isomer overlap is still possible. These bicyclic hydrocarbons have been noted to exhibit antioxidant activity and are also likely to regulate the oxidative stability of the essential oil.

The major component of the chromatogram was o-cymene, with the retention time of 6.547 min (32.95 percent). Its Kovats index (~1,026) and several overlapping spectra are all evidence of strong assignment, but it is not possible to exclude the possibility of co-eluting with other tetramethyl-benzene derivatives which are also well-established in thyme oils as precursors to thymol and carvacrol biosynthesis.

Another abundant monoterpene was gamma-terpinene (RT 7.109 min, 16.85%, KI ~ 1,059). It is validated by its great match scores (80194), and by its sharp peak profile. This compound along with cymene is the major intermediate in the phenolic monoterpene pathway especially in thymol-abundant chemotypes.

Other molecules that were identified were minor oxygenated monoterpenes such as p-menth-8-en-1-ol isomers (RT 7.378 min, 1.47%, KI ~ 1,088) and terpinolene (RT 7.620 min, 0.63%, KI ~ 1,088). There was high confidence (>90%)-matchedness in both, yet because of stereoisomeric overlap, accurate structural assignments are made difficult. These compounds are proven to have the effect on aroma complexes and can have antimicrobial properties.

Terpinen-4-ol (RT 9.403 min, 1.32%, KI ~ 1,177) was confidently identified (match 89–94%). It is one of the major oxygenated derivatives of alcohol in thyme and related oils, which have been known to have a high level of antibacterial and antifungal effects.

Later in the run were the phenolic derivatives. 2,3,5,6-tetramethylphenol (RT 11.956 min, 1.56%, KI ~ 1,260) was detected as a minor phenolic component. More importantly, thymol emerged at 12.199 min with a significant contribution (11.30%). Its Kovats index (~1,290) is highly consistent with literature data, and although the spectral quality (87%) indicates possible overlap with carvacrol, thymol is strongly supported as the chemotypic marker of this oil. Thymol is the principal bioactive agent in thyme, with broad-spectrum antimicrobial, antioxidant, and anti-inflammatory activities.

Taken together, the GC–MS analysis demonstrates that the essential oil of *Thymus vulgaris* in this study is predominantly composed of monoterpene hydrocarbons (o-cymene, gamma-terpinene) and oxygenated monoterpenes (thymol, terpinen-4-ol). The high proportion of cymene and gamma-terpinene indicates a thymol chemotype, where these hydrocarbons act as biosynthetic precursors to the phenolic marker. The detection of minor bicyclic terpenes and alcohols further enriches the compositional profile but requires confirmation to exclude analytical artefacts or co-elutions.

### 3.2 MLST profile interpretation

Multilocus sequence typing (MLST) analysis identified the isolate as belonging to Sequence Type (ST) 1104. This classification was based on the unique combination of alleles from seven conserved housekeeping genes: gltA (allele 2), gyrB (allele 21), gdhB (allele 12), recA (allele 32), cpn60 (allele 26), gpi (allele 106), and rpoD (allele 5). ST1104 is a unique clonal lineage that is determined by the particular allelic profile. MLST is a molecular typing technique that is highly popular and enables to compare bacterial isolates using sequence data, to help in the exploration of the evolutionary relationship, outbreak monitoring, and international epidemiological surveillance.

### 3.3 *A. baumannii* strain muks92 Elements, and plasmids

Genomic data on the *Acinetobacter baumannii* strain muks92 indicated a wide array of antimicrobial resistance genes, mobile genetic elements (MGEs) and plasmid replicons. Four different genes were found to be *β*-lactamases, namely blaOXA-90 and blaOXA-72 which mediate carbapenem resistance and blaADC-25 and blaCARB-14 which mediate cephalosporinase and broad-spectrum ß-lactamase activities respectively. Such genes were present on different scaffold and were highly related to reference sequences.

In the case of aminoglycoside resistance, three significant genes were identified and included in amikacin resistance and gentamicin resistance armA, a 16S rRNA methyltransferase. Also, aac(6 3 -1)-Ian and aadA5 are involved in tobramycin and streptomycin resistance, respectively.

In aminoglycoside resistance, three important genes were identified as armA is a 16S rRNA methyltransferase which gives resistance to several aminoglycosides such as amikacin and gentamicin. Also, aac(6´)- I and aadA5 mediate resistance to tobramycin and streptomycin respectively. Macrolide resistance was conferred by mph(E) and msr(E), which mediate efflux and antibiotic inactivation mechanisms.

A biocide resistance gene, qacE, known for reducing susceptibility to quaternary ammonium compounds and disinfectants, was also identified, albeit with slightly lower coverage.

The genome harbored several insertion sequences (IS elements), including ISEc29, ISAba1, ISAba13, ISAba21, IS17, and IS26, affiliated with IS families IS3, IS4, IS5, and IS6. These MGEs are known to facilitate horizontal gene transfer and genome rearrangements. The detection of both forward and reverse strand orientations suggests active mobilization within the genome.

Finally, a plasmid replicon of the IncA/C2 type was detected, commonly associated with multidrug resistance in Enterobacteriaceae. Its presence suggests a high potential for interspecies gene transfer (see Table 4).

**Table 4:** Elements, and plasmids in *A. baumannii* strain muks92.

Category	Gene/element	Phenotype/type	Scaffold	Position (bp)	Coverage	Identity (%)	Strand/notes
MGEs (IS)	ISEc29	IS4 family (IS10 group)	Scaffold56_1	4,566-5,890	100%	100.00	Reverse
	IS1006	IS6 family	–	156-974	100%	99.88	Reverse
	ISAba1	IS4 family	Scaffold95_1	1-1,180	100%	100.00	Forward
	ISAba13	IS5 family (IS903 group)	Scaffold6_1	17,024-18,062	100%	100.00	Reverse
	ISAba21	IS3 family	Scaffold76_1	2,004-3,277	100%	100.00	Forward
	IS17	IS5 family (IS903 group)	Scaffold96_1	1-1,039	99.9%	95.19	Reverse
	IS26	IS6 family	Scaffold99_1	1-820	100%	99.63	Reverse
Plasmids	IncA/C2	Plasmid replicon (Enterobacteriaceae)	Scaffold41_1	15,130-15,546	100%	100.00	Plasmid-associated

### 3.4 Distribution of antibiotic resistance genes in *A. baumannii* strain muks92

Whole-genome analysis of *Acinetobacter baumannii* strain muks92 revealed the presence of multiple antibiotic resistance genes distributed across various genomic scaffolds. The strain harbored several *β*-lactamase-encoding genes, including blaOXA-90 and blaOXA-72, both showing 100% identity and complete coverage, located on Scaffold12_1 and Scaffold51_1, respectively. Additionally, blaADC-25, an AmpC-type *β*-lactamase, was identified on Scaffold34_1 with 99.91% coverage and 96.27% identity, while blaCARB-14, associated with resistance to ampicillin, amoxicillin, and piperacillin, was found on Scaffold84_1. Aminoglycoside resistance determinants included armA, aac(6′)-Ian, and aadA5, conferring resistance to a broad spectrum of aminoglycosides such as gentamicin, tobramycin, and streptomycin; these were located on Scaffold56_1 and Scaffold41_1. Sulfonamide resistance genes sul1 and sul2 were also detected, with sul2 present (see Table 5).

**Table 5:** Distribution of antibiotic resistance genes in *A. baumannii* strain muks92.

Category	Gene/element	Phenotype/type	Scaffold	Position (bp)	Coverage	Identity (%)	Strand/notes
β-lactamases	blaOXA-90	β-lactam (unknown)	Scaffold12_1	86,817-87,641	100%	100.00	–
	blaOXA-72	β-lactam (unknown)	Scaffold51_1	14,423-15,250	100%	100.00	–
	blaADC-25	β-lactam (AmpC-type)	Scaffold34_1	1,224-73	99.91%	96.27	–
	blaCARB-14	Ampicillin, amoxicillin, piperacillin	Scaffold84_1	1,520-624	100%	99.89	–
Aminoglycosides	armA	Gentamicin, tobramycin, amikacin, netilmicin, isepamicin	Scaffold56_1	6,500-7,273	100%	100.00	–
	aac(6′)-Ian	Tobramycin, amikacin, netilmicin, dibekacin, sisomicin	Scaffold41_1	648-76	100%	100.00	–
	aadA5	Spectinomycin, streptomycin	Scaffold56_1	11,966-12,754	100%	100.00	–
Sulfonamides	sul1	Sulfamethoxazole	Scaffold56_1	10,618-11,457	100%	100.00	–
	sul2	Sulfamethoxazole	Multiple	2,371-2,933	~69%	100.00	Three copies
Macrolides/MLS	mph€	Erythromycin	Scaffold56_1	1,786-2,670	100%	100.00	–
	msr€	Quinupristin, azithromycin, erythromycin, pristinamycin	Scaffold56_1	2,726-4,201	100%	100.00	–
Biocides	qacE	Benzalkonium chloride, chlorhexidine, ethidium bromide	Scaffold56_1	11,517-11,798	84.68%	100.00	–
MGEs (IS)	ISEc29	IS4 family (IS10 group)	Scaffold56_1	4,566-5,890	100%	100.00	Reverse
	IS1006	IS6 family	–	156-974	100%	99.88	Reverse
	ISAba1	IS4 family	Scaffold95_1	1-1,180	100%	100.00	Forward
	ISAba13	IS5 family (IS903 group)	Scaffold6_1	17,024-18,062	100%	100.00	Reverse
	ISAba21	IS3 family	Scaffold76_1	2,004-3,277	100%	100.00	Forward
	IS17	IS5 family (IS903 group)	Scaffold96_1	1-1,039	99.9%	95.19	Reverse
	IS26	IS6 family	Scaffold99_1	1-820	100%	99.63	Reverse
Plasmids	IncA/C2	Plasmid replicon (Enterobacteriaceae)	Scaffold41_1	15,130-15,546	100%	100.00	Plasmid-associated

### 3.5 Virulence factor genes diffusion in *A. baumannii* strain muks92

Whole genome sequencing of *A. baumannii* strain muks92 revealed highly diverse expression of virulence-associated genes spread over several scaffolds each of which is related to major pathogenic processes such as biofilm formation, iron acquisition, adhesion and antibiotic efflux.

The most notable ones comprised the elements of the AdeFGH efflux system adeF, adeG, and adeH, situated at Scaffold13_1, encode a membrane-fusion protein, a multidrug efflux pump, and an outer membrane constituent respectively, and all of which have over 97% sequence identity. On Scaffold9_1, a bap gene, which is associated with biofilm maturation and persistence, was found with an identity of 99.27%.

The genes that are associated with acquisition of iron and siderophore biosynthesis were most abundant on Scaffold34_1. These contained barA and barB that encode parts of the ABC-type siderophore efflux system and bas gene cluster (basA through basJ), which are involved in acinetobactin biosynthesis. It is important to note, that the genes bauB to bauF encode proteins of the ferric-siderophore ABC transport system, which is essential during the iron uptake under low iron conditions.

The two-component system that is involved in biofilm formation and surface adhesion is encoded by the regulatory genes bfmR and bfmS, which are located on Scaffold4_1. Equally, csu operon (csuA, csuA/B, csuB, csuC, csuD, csuE) present on Scaffold50_1 codes Csu pilus assembly system, which is important in the initial adhesion to abiotic surface- essential to catheter-related infections.

Scaffold11_1 had the highly conserved (98.97) ompA gene of the outer membrane which has been known to mediate host-cell adhesion, immune evasion, and serum resistance. Moreover, the Scaffold16_1 pgaA-D gene cluster provides the machinery needed to synthesize and export poly- 2,1,6 -N -acetyl-D-glucosamine, an exopolysaccharide that is vital in the integrity of biofilms.

There were also enzymes that confer membrane disruption and virulence such as plc (expressed on Scaffold17_1 and Scaffold8_1) and plcD (expressed on Scaffold39_1) that encode phospholipases that mediate tissue invasion and lysis of host cells.

This virulome landscape underscores the multifactorial pathogenic potential of *A. baumannii* strain muks92, characterized by robust biofilm capability, efficient iron scavenging systems, surface adhesion mechanisms, and resistance-associated efflux pumps—all of which may contribute to its clinical resilience and environmental persistence (see Table 6).

**Table 6:** Distribution of virulence factors genes in *A. baumannii* strain muks92.

No.	Virulence gene	Contig	Identity (%)	Position	Functional annotation
1	adeF	Scaffold13_1	97.87	10775.0.11992	Membrane-fusion protein
2	adeG	Scaffold13_1	97.33	7589.0.10768	Cation/multidrug efflux pump
3	adeH	Scaffold13_1	98.34	6128.0.7576	Outer membrane protein
4	bap	Scaffold9_1	99.27	119041.0.124281	Biofilm-associated protein
5	barA	Scaffold34_1	97.14	11862.0.13472	Siderophore efflux system (ABC superfamily)
6	barB	Scaffold34_1	97.81	10270.0.11865	Siderophore efflux system (ABC superfamily)
7	basA	Scaffold34_1	95.18	30804.0.32651	Acinetobactin biosynthesis protein
8	basB	Scaffold34_1	95.47	28637.0.30733	NRPS with condensation & peptidyl carrier protein domains
9	basC	Scaffold34_1	98.02	20653.0.21963	Acinetobactin biosynthesis protein BasC
10	basD	Scaffold34_1	96.88	17756.0.20605	Acinetobactin biosynthesis protein BasD
11	basF	Scaffold34_1	97.59	14987.0.15856	Aryl carrier protein BasF
12	basG	Scaffold34_1	97.83	13718.0.14869	Acinetobactin biosynthesis protein BasF
13	basH	Scaffold34_1	96.73	9464.0.10197	NRPS thioesterase BasH
14	basI	Scaffold34_1	96.43	8698.0.9453	Phosphopantetheinyl transferase (BasI)
15	basJ	Scaffold34_1	97.95	7404.0.8573	Acinetobactin biosynthesis protein BasJ
16	bauB	Scaffold34_1	98.45	24443.0.25411	Ferric siderophore ABC transporter, periplasmic binding protein
17	bauC	Scaffold34_1	96.41	26185.0.27132	Ferric siderophore ABC transporter, permease protein BauC
18	bauD	Scaffold34_1	96.01	27146.0.28073	Ferric siderophore ABC transporter, permease protein BauD
19	bauE	Scaffold34_1	99.74	25418.0.26188	Ferric siderophore ABC transporter, ATP-binding protein BauE
20	bauF	Scaffold34_1	96.75	33044.0.33904	Siderophore-interacting protein
21	bfmR	Scaffold4_1	99.30	55979.0.56695	Biofilm-controlling response regulator
22	bfmS	Scaffold4_1	98.91	56728.0.58377	Signal transduction histidine kinase
23	csuA	Scaffold50_1	97.32	7907.0.8353	Csu pilus subunit CsuA
24	csuA/B	Scaffold50_1	98.70	7194.0.7730	Csu pilus major pilin subunit CsuA/B
25	csuB	Scaffold50_1	99.04	8359.0.8877	Csu pilus subunit CsuB
26	csuC	Scaffold50_1	98.56	8871.0.9704	Csu pilus chaperone protein CsuC
27	csuD	Scaffold50_1	97.64	9701.0.12199	Csu pilus usher protein CsuD
28	csuE	Scaffold50_1	95.78	12196.0.13215	Csu pilus tip adhesin CsuE
29	entE	Scaffold34_1	97.36	15874.0.17502	NRPS adenylate-forming enzyme (acinetobactin synthesis)
30	ompA	Scaffold11_1	98.97	95796.0.96866	Outer membrane protein OmpA
31	pgaA	Scaffold16_1	98.98	37498.0.38871	Poly-β-1,6 N-acetyl-D-glucosamine export porin PgaA
32	pgaB	Scaffold16_1	98.47	39900.0.41729	Poly-β-1,6-N-acetyl-D-glucosamine N-deacetylase PgaB
33	pgaC	Scaffold16_1	97.92	41729.0.42976	Poly-β-1,6 N-acetyl-D-glucosamine synthase
34	pgaD	Scaffold16_1	97.63	42973.0.43437	Poly-β-1,6-N-acetyl-D-glucosamine biosynthesis protein PgaD
35	plc	Scaffold17_1	95.67	25219.0.27388	Phospholipase C
36	plc	Scaffold8_1	96.63	118455.0.120620	Phospholipase C
37	plcD	Scaffold39_1	99.32	21498.0.23123	Phosphatidylserine/glycerophosphate/cardiolipin synthase

### 3.6 Distribution of phage and crisper genes in XDR *Acinetobacter baumannii* strain: muks92

No CRISPR elements or prophage sequences were identified in the genome of the extensively drug-resistant *Acinetobacter baumannii* strain muks92.

#### 3.6.1 cgMLST

**Figure 3:**
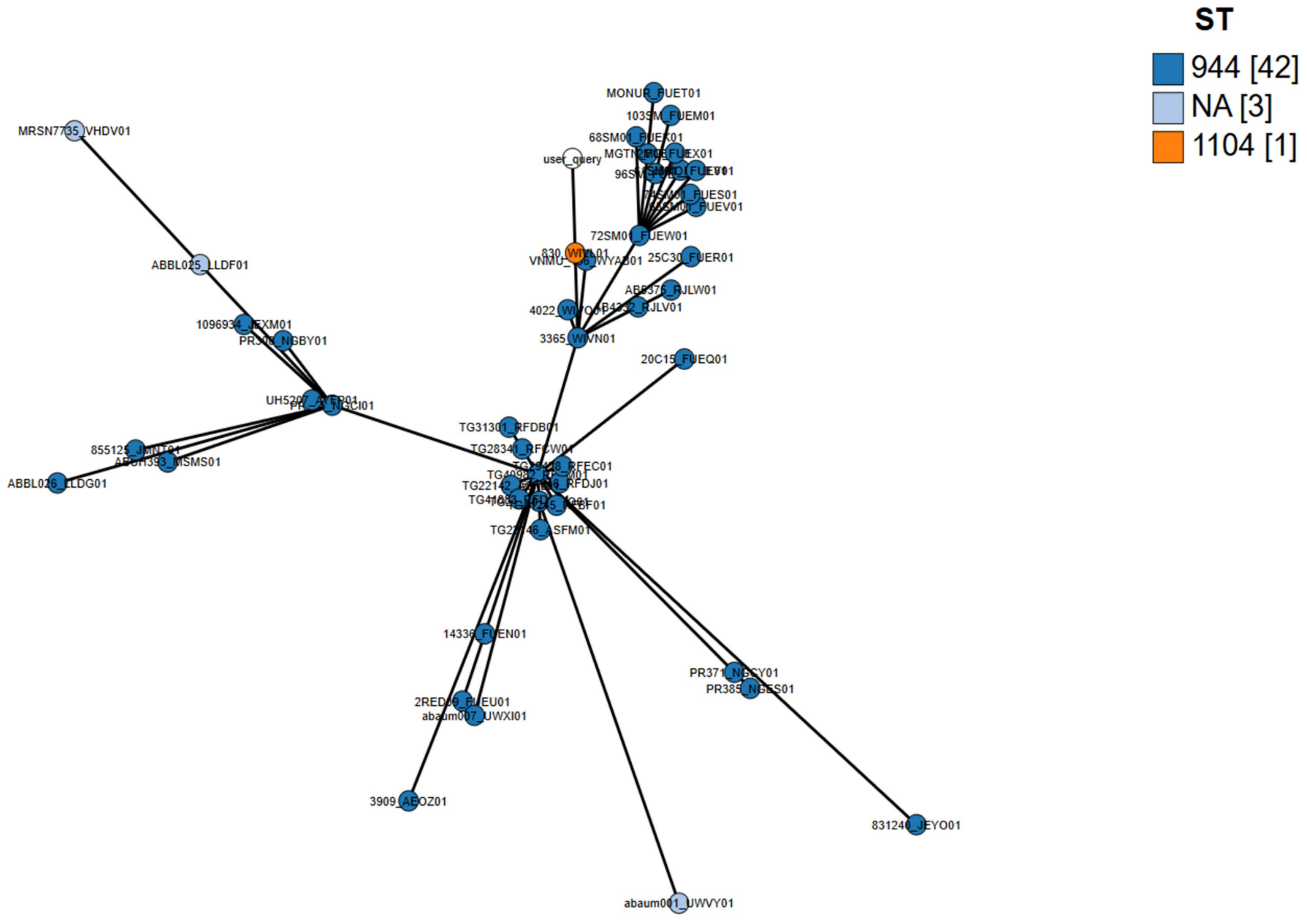
Global relatedness of *A. baumannii* isolates by cgMLST (nodes by ST).

Supplementary Figure and Figure 3 show the set is dominated by *A. baumannii* ST944 (with one ST1104), sampled from multiple body sites in Brazil, Ukraine, Switzerland, the USA (AZ/OH/MD/OH), and Georgia (Tbilisi) from 2006 to 2018, indicating long-term, multi-continental persistence of a single high-risk lineage. AMR backbones are highly conserved—blaADC-25 plus blaOXA-90 in nearly all—overlaid by modular acquisitions: OXA-200 in a 2012 Arizona tracheal-aspirate cluster, OXA-72 in Brazil/Ukraine, and sporadic OXA-23/58/545; CTX-M-124 appears in Brazil/Ukraine/Georgia. Aminoglycoside resistance is pervasive (aac(3)-IIa, aac(6′)-Ian, ant(2″)-Ia, aph(3′)-Ia) with frequent armA; macrolide (mph(E)/msr(E)), phenicol (floR/catA1), and sul genes vary by isolate. Virulence modules are remarkably stable—acinetobactin bas/bau, csu pili, bap and pgaABCD biofilm genes, ompA, adeFGH efflux, and bfmRS—with quorum-sensing (abaI/abaR) present in a subset. cgMLST distances span 54–423 alleles: the three 2018 Tbilisi isolates form a tight cluster (54–84), the OXA-200 Arizona group sits mid-range (~158–166), and older/geo-distant entries diverge further—consistent with recent local transmission events nested within a widely disseminated ST944 complex.

### 3.7 Treatment of biofilm with *Thymus vulgaris*

**Figure 4:**
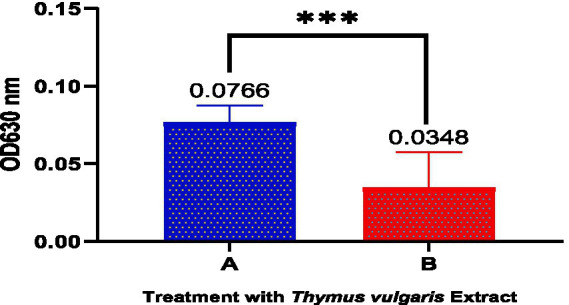
Treatment of biofilm with *Thymus vulgaris*. Bar graph showing a significant reduction in biofilm biomass (OD630 nm) after treatment with *T. vulgaris* essential oils **(B)** compared to the untreated control **(A)**.

Figure 4 and Table 7 quantitative analysis of biofilm inhibition following treatment with *Thymus vulgaris* essential oils revealed a significant reduction in biofilm biomass. Group A (untreated control) exhibited a mean absorbance of 0.07660 ± 0.01098, indicating substantial biofilm formation. In contrast, Group B (treated with *T. vulgaris*) showed a markedly lower mean absorbance of 0.03480 ± 0.02267, suggesting effective inhibition of biofilm development. The standard error of the mean for Groups A and B were 0.003471 and 0.007168, respectively. Statistical comparison between the two groups yielded a highly significant *p*-value of 0.0002 (***), demonstrating that *T. vulgaris* essential oils significantly disrupted biofilm formation in the tested *Acinetobacter baumannii* strain.

**Table 7:** Treatment of biofilm with *Thymus vulgaris*.

Statistical measure	A	B
Mean	0.07660	0.03480
Std. deviation	0.01098	0.02267
Std. error of mean	0.003471	0.007168
*p*-value	0.0002***	

### 3.8 Impact of *Thymus vulgaris* on extended-spectrum *β*-lactamase (ESBL)-producing isolates

**Figure 5:**
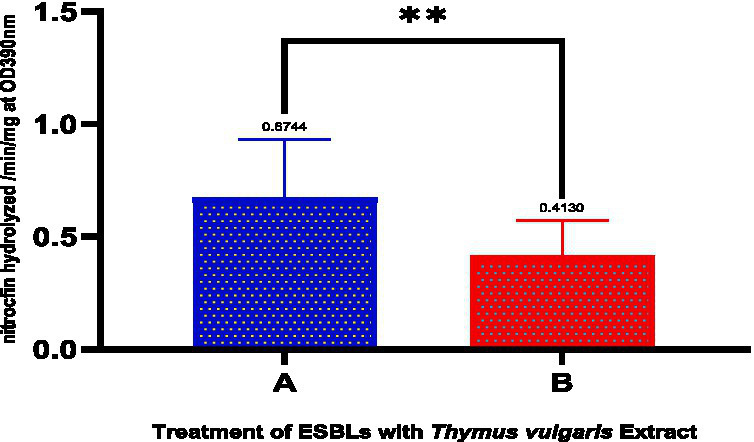
Effect of *Thymus vulgaris* essential oils on the activity of extended-spectrum β-lactamase (ESBL)-producing isolates. The graph compares the hydrolysis of nitrocefin (OD390 nm/min) between untreated **(A)** and *Thymus vulgaris*-treated **(B)** groups. A significant reduction (**p < 0.01) in ESBL activity was observed following treatment with the essential oils.

Figure 5 and Table 8 depict the action of extended-spectrum *β*-lactamase (ESBL)-producing isolates treated with *Thymus vulgaris* extract indicated there was a considerable inhibitory action as seen through the comparative statistical analysis. The mean value of the pre-treatment and post-treatment reduced significantly by 0.6744 to 0.4130 implying that the extract was effective in inhibiting the ESBL activity by almost 39 percent. The decreasing standard deviation (0.2589 to 0.1596) and standard error of the mean (0.08186 to 0.05047) demonstrate the reason why consistency and precision have improved in the measurements after treatment. Notably, the statistical test showed p-value of 0.0017, which supports the fact that the reduction in question is extremely significant and it could not have possibly occurred due to chance. Such a high level of significance (*p* < 0.01) highlights the method-reproducible nature and strength of the inhibitory effect. Phytochemicals, including thymol, carvacrol, and gamma-terpinene, which are known to disrupt bacterial resistance mechanisms, either by disrupting -lactamase enzymatic sites or by altering resistance expression pathways, can be attributed to the activity, biologically. Taken together, these results represent a solid piece of evidence that *T. vulgaris* has a demonstrable statistically confirmed effect on the ESBL-producing bacteria, and can be used as a natural antimicrobial agent against pathogenic organisms resistant to antibiotics.

**Table 8:** Treatment of ESBLS with *Thymus vulgaris*.

Statistical measure	A (before treatment)	B (after treatment)
Mean	0.6744	0.4130
Std. deviation	0.2589	0.1596
Std. error of mean	0.08186	0.05047
*p-value*	0.0017	

### 3.9 Phylogenetic tree of *Acinetobacter baumannii* isolate muks92 based on a 16S rRNA

**Figure 6:**
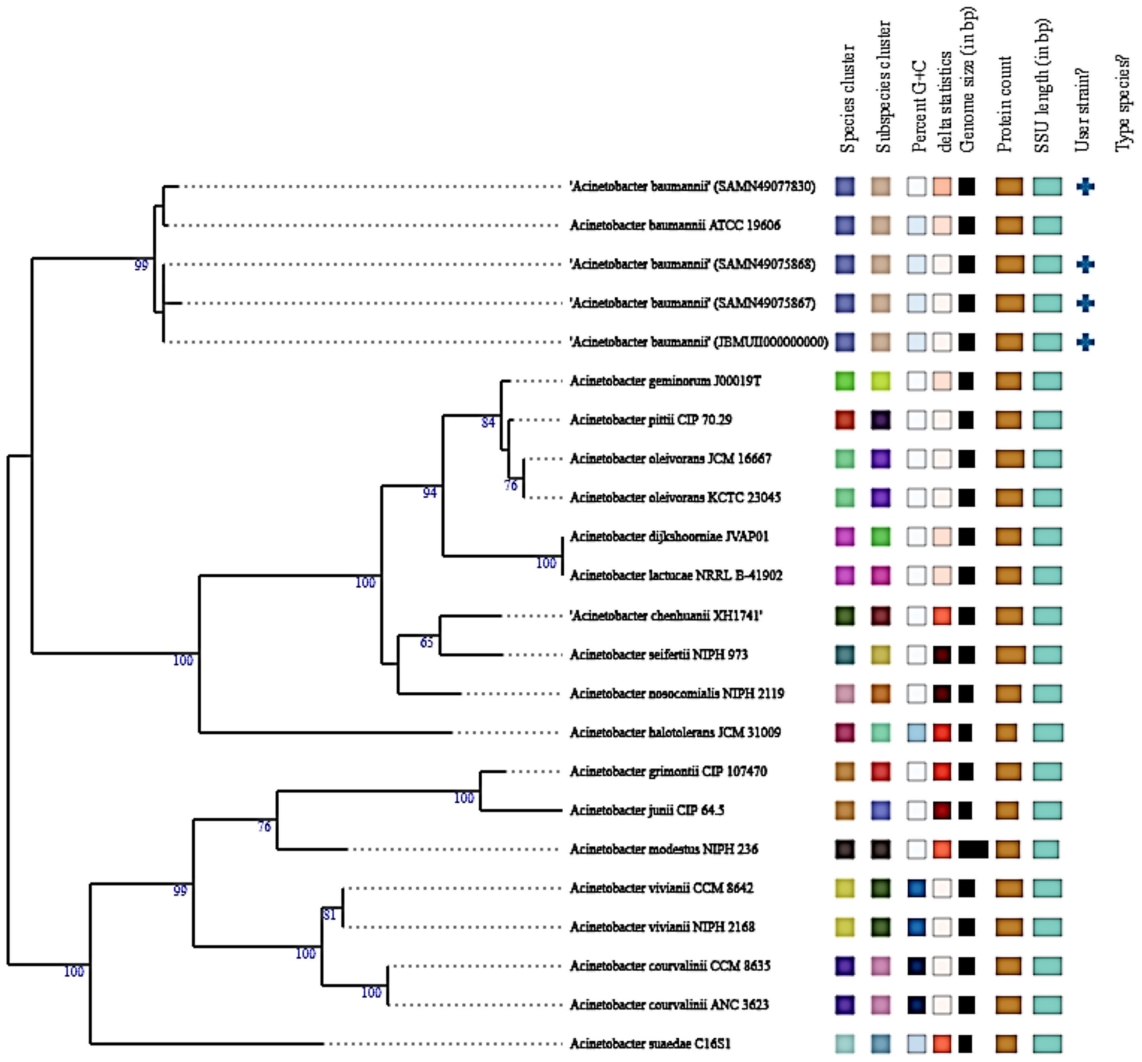
Phylogenetic tree of *Acinetobacter baumannii* isolate muks92 based on A 16S rRNA. The evolutionary relationship of isolate muks92 is shown in comparison with reference Acinetobacter species. Bootstrap values (≥50%) are indicated at the branching points, supporting the clustering of muks92 within the *A. baumannii* clade.

Figure 6 represents Whole-genome-based phylogenetic analysis confirmed the precise taxonomic placement of the *Acinetobacter baumannii* isolate muks92 (accession: JBMUII000000000). The maximum likelihood tree revealed that muks92 clustered closely with well-established *A. baumannii* reference strains, including ATCC 19606 and SAND440973830, with a bootstrap support of 99%, indicating strong evolutionary relatedness.

The isolate shared conserved genomic features with these reference strains, such as:

- A 16S rRNA gene length of approximately 1,500 bp,
- GC content around 39%,
- Genome size ranging between 3.9 to 4.1 Mbp.

No evidence suggested hybrid lineage or interspecies recombination, and the isolate aligned within the clade containing type strains, as indicated by the corresponding “+” symbol. These results confirm that muks92 belongs to the *A. baumannii* sensu stricto lineage, with no deviation in core taxonomic markers.

### 3.10 Phylogenetic tree of *Acinetobacter baumannii* isolate muks92 based on whole genome

**Figure 7:**
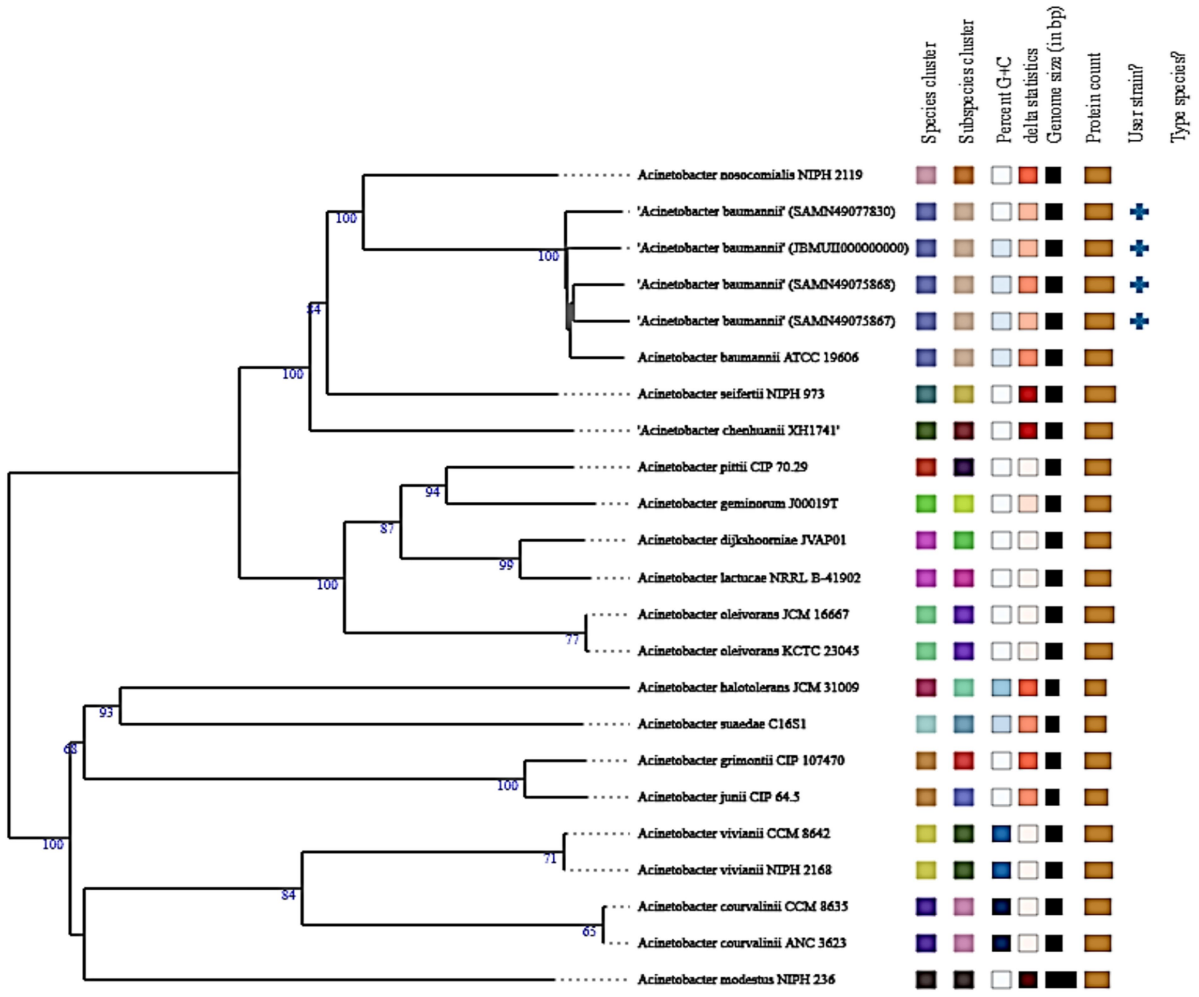
Phylogenetic tree of *Acinetobacter baumannii* isolate muks92 based on whole genome. The tree illustrates the evolutionary placement of the muks92 isolate in relation to reference *Acinetobacter* species. Bootstrap support values are indicated at branching nodes, confirming the clustering of muks92 within the *A. baumannii* lineage and its close relationship to clinically relevant strains.

Figure 7 represents whole-genome phylogenetic reconstruction was performed to determine the evolutionary position of *A. baumannii* isolate muks92 in relation to other members of the Acinetobacter genus. The resulting tree clearly places muks92 within a highly supported clade of *A. baumannii* strains, indicating strong genomic relatedness to known clinical and reference isolates. This clade includes well-characterized genomes such as ATCC 19606 and several annotated isolates from GenBank (e.g., GCA_004007895.1, GCA_004007885.1, GCA_004007865.1), all of which form a distinct cluster with high bootstrap values (100%), signifying robust phylogenetic support.

The close association of muks92 with this cluster underscores its genomic affiliation with pathogenic strains of *A. baumannii*, many of which are implicated in multidrug-resistant nosocomial infections. This relationship suggests that muks92 shares common evolutionary ancestry, conserved genomic content, and potentially similar resistance and virulence mechanisms with other globally disseminated strains. It is also found to be in this lineage and this, combined with its genotypic designation of the Sequence Type (ST) 1104 and hallmark resistance genes and virulence determinants detected in its genome, also puts it in this lineage.

The phylogenetic tree, on the contrary, indicates well-defined boundaries of genome between *A. baumannii* and other species of Acinetobacter *A. nosocomialis*, *A. pittii*, *A. seifertii*, *A. johnsonii*, *A. calcoaceticus*, and *A. lwoffii*, which are distinct lineages. These species clusters are characterized by a long branch length and a high-confidence bootstrap value which shows that there is a great divergence among the species. This isolation shows the genomic diversity of the genus and allows proper demarcation of the species by whole-genome measures.

As well, the phylogenetic tree combines phenotypic and genotypic characteristics, such as the content of plasmids, the profiles of resistance genes, and the types of sequences, as can be seen in the metadata provided in colors. The similarity of these features in closely related *A. baumannii* isolates can indicate a common horizontal gene transfer event and evolution forces that shaped the resistome and virulome of the lineage. Specifically, the association of muks92 with plasmid-carrying, resistance-encoded strains is an indicator of its clinical importance and the possibility of human involvement in the process of antimicrobial resistance transmission.

In general, the location of *A. baumannii* muks92 in a clearly delimited pathogenic cluster supports the notion that it is a clinically relevant strain, which is genetically related to epidemic clones. The analysis confirms its classification as a species, as well as gives a genomic framework in explaining its epidemiology, resistance, and evolution in the *Acinetobacter* genus.

### 3.11 Molecular docking

**Figure 8:**
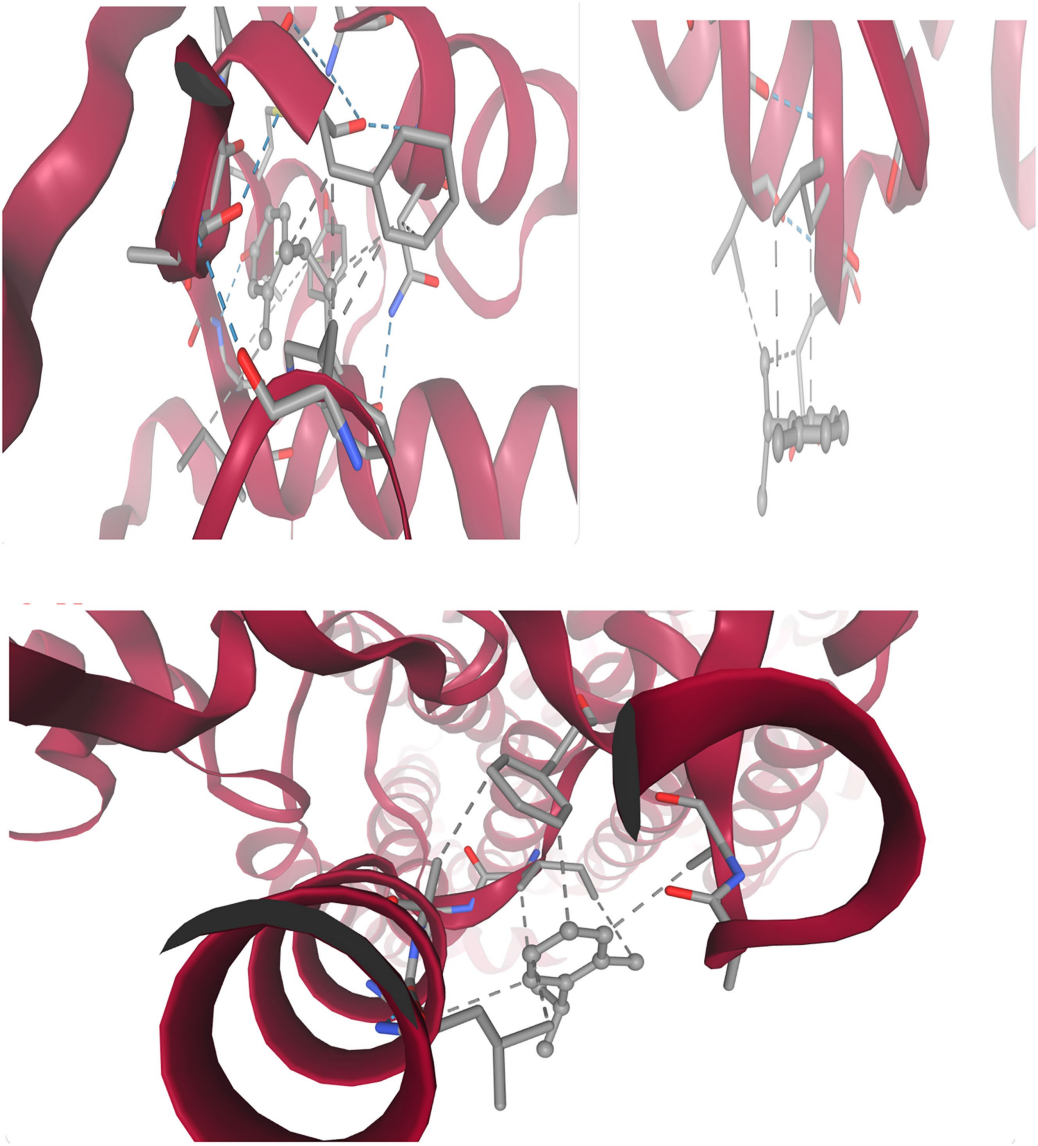
Interaction of o-cymene with target proteins of *Acinetobacter baumannii*: **(A)** 6T1H, **(B)** 5HM6, **(C)** 8YR0.

Figure 8 and Table 9 present integrated structural interpretation of o-cymene binding.

**Table 9:** Key interacting residues and binding energies of *o-Cymene* docked with resistance-associated proteins of *Acinetobacter baumannii*.

Target (PDB)	Key hydrophobic residues	Aromatic π–π residues	Polar/H-bond contributors	Best docking affinity (kcal·mol^−1^)	Affinity range (kcal·mol^−1^)
β-lactamase (6T1H)	Leu, Val, Ile	Phe, Tyr	Ser, Thr, Asn, Gln, Lys, Arg, Asp., Glu	−6.052	−6.052 to −4.816
Quorum-sensing or biofilm-controlling response regulator (5HM6)	Leu152, Ile156, Val110	Phe108, Tyr160	Ser112, Thr114, Gly111, Ala113	−3.412	−3.412 to −2.970
Efflux pump (8YR0)	Leu272, Ile276, Val280, Ala304, Leu308	Phe310, Tyr315	Ser278, Thr312, Gly309	−3.920	−3.920 to −2.693

#### 3.11.1 Deep structural interaction analysis of o-cymene with 6T1H, 5HM6, and 8YR0

The interaction of *o*-Cymene with bacterial resistance proteins demonstrates how this small hydrophobic aromatic compound adapts to structurally distinct binding pockets, relying on a balance of nonpolar encapsulation, aromatic *π*–π stabilization, and limited polar contacts.

Within *β*-lactamase (6T1H), *o*-Cymene is situated in a compact hydrophobic cleft bordered by *α*-helices, where it achieves the strongest binding among the tested proteins. The isopropyl substituent of the ligand is buried in a nonpolar shell formed by leucine, valine, and isoleucine residues, while the aromatic ring is braced by phenylalanine and tyrosine through *π*–π stacking. Importantly, the polar face of the cavity contributes additional stabilization: hydroxyl-bearing serine and threonine establish weak hydrogen bonds, while asparagine and glutamine amides extend polarity into the cleft. Charged residues, including lysine, arginine, aspartic acid, and glutamic acid, enhance electrostatic complementarity, anchoring the ligand. This is a network of diverse interactions that clarifies the optimal affinity of –6.052 kcal-mol^–1^ which is a sense of strong complementarity as well as high selectivity.

Conversely, quorum-sensing protein (5HM6) is a comparatively shallow pocket, and *o*-Cymene fits in its groove in parallel with the helical axis. In this case, the hydrophobic stabilization is mainly provided by leucine-152, isoleucine-156, and valine-110 and these amino acids make a lipophilic pocket around the isopropyl moiety. Phenylalanine-108 and tyrosine-160 bracket the benzene ring with an edge-to-face and T-shaped geometry respectively but the interactions are not as strong as 2-lactamase. Potential weak hydrogen-bond interactions are contributed by serine-112 and threonine-114, which are supported by glycine-111 and alanine-113 backbone carbonyls. The docking affinity is not so high because of no deep insertion or high polarity, and its best energy is at –3.412 kcal/mol^–1^, which means there is loose binding, but without specificity.

Lastly, the efflux pump (8YR0) has a longer transmembrane tunnel, which hydrophobic substrates are comfortable with, with *o*-Cymene fitting well lengthwise, and enclosed by leucine-272, isoleucine-276, valine-280, alanine-304, and leucine-308 to form a nonpolar cage. Phenylalanine-310 and tyrosine-315 clamp the aromatic ring, which strengthens π-stacking and T-shaped stabilization. Secondary anchoring is provided by polar contributions of serine-278, threonine-312 and carbonyl of the glycine-309 backbone. Intermediate stabilization is observed at the docking energies (best –3.920 kcal which are weak –3.920 mol^–1^) but is weaker than the quorum-sensing regulator (but stronger than 3-lactamase) as is expected by the natural promiscuity of the tunnel with lipophilic ligands.

This comparative docking paper points out a pyramid of preferences in the *o*-Cymene binding. The highest affinity is observed in the *β*-lactamase pocket (6T1H) with a cooperative network of hydrophobic, aromatic, and polar contacts, indicating that o-Cymene may be an inhibitor and disrupt enzymatic hydrolysis of 6T1H-binding 6T1H-binding 6T1H-binding 6T1H-binding 6T1H-binding 6T1H-binding 6T1H-binding 6T1H-binding 6T1H-binding 6T1 The binding of the efflux pump (8YR0) is intermediate (indicating nonspecific jamming of hydrophobicity in its transport tunnel), which can temporarily prevent the efflux of drugs. The quorum-sensing regulator (5HM6), in contrast, displays the lowest binding which is expected to be due to a shallow groove that offers little specificity. In general, the results indicate that *o*-Cymene has the highest potential as a *β*-lactamase-inhibitor, and additional modulatory effect on efflux and quorum-sensing pathways, which is consistent with the suggested proposal to counter bacterial resistance mechanisms.

#### 3.11.2 Resistance-associated protein targets o-cymene deep interaction analysis

**Figure 9:**
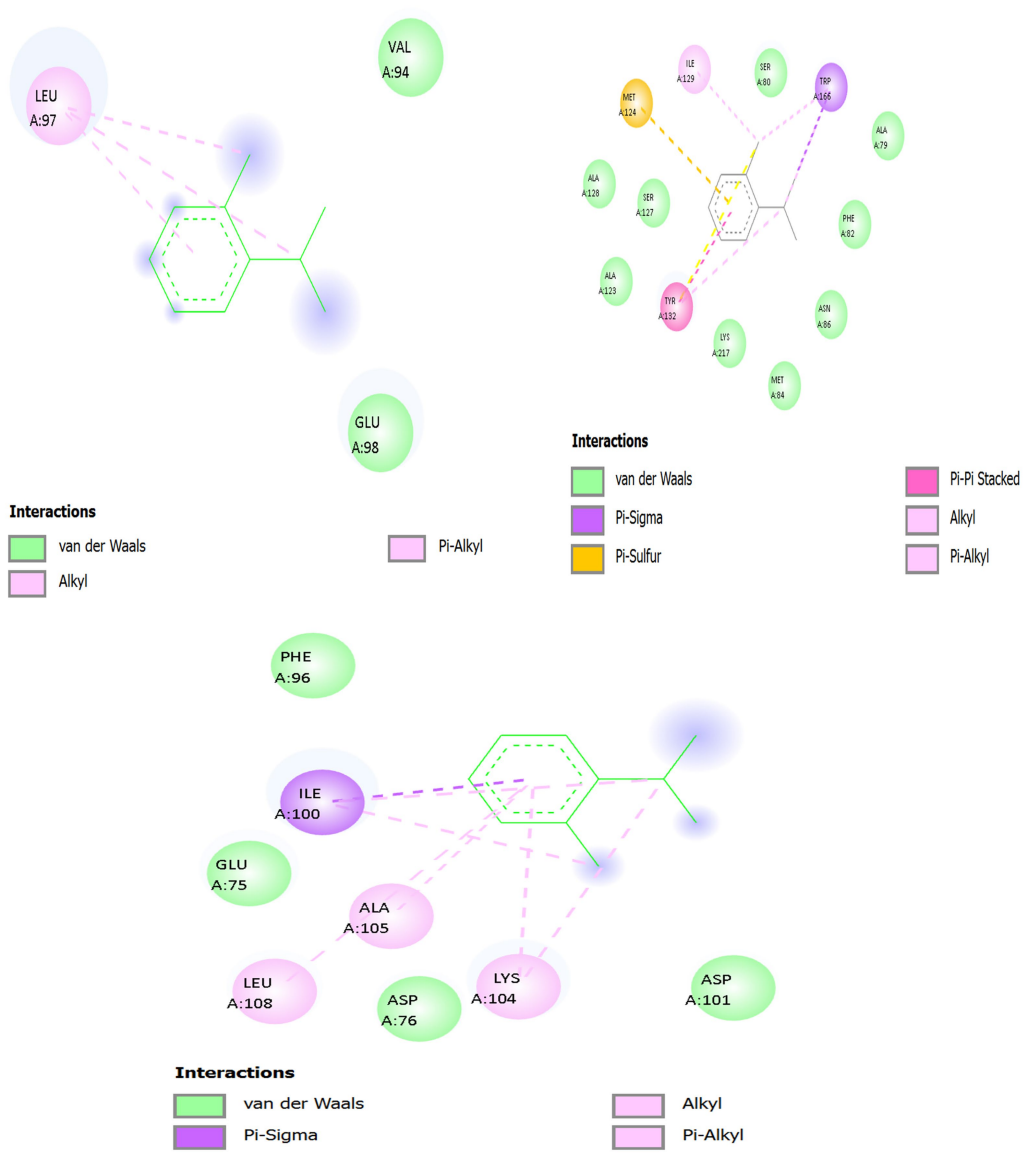
2D interaction of o-cymene with target proteins of *Acinetobacter baumannii*: **(A)** 5HM6, **(B)** 6T1H, **(C)** 8YR0.

Figure 9 and Table 10 show the docking of the small hydrophobic aromatic hydrocarbon *o*-Cymene on bacterial resistance-associated proteins indicates a uniform dependency on van der Waals complementarity, hydrophobic encapsulation, and *π* -alkyl stabilization. It has a non-polar nature and a planar benzene framework that can be easily accommodated into various cavities and transport channels of the enzyme, indicating its possible modulatory activity in bacterial resistance.

**Table 10:** 2D Interaction summary of o-cymene with protein targets.

Target protein (PDB)	Principal hydrophobic contacts	Aromatic / π–alkyl interactions	Polar/electrostatic influence	Interaction outcome
Biofilm-controlling response regulator (5HM6)	Leu A:97, Val A:94	None significant (ring anchored to Leu aliphatic side chain)	Glu A:98 (negative charge stabilizing π-system)	Aromatic core anchored by leucine; stable hydrophobic embedding near catalytic site.
β-lactamase (6T1H)	Met A:127, Met A:129, Ile A:173, Ile A:174	Phe A:66, Tyr A:105 (π–alkyl)	Ala A:128, Ala A:130, Lys A:131, Ser A:132 (structural confinement)	Tight hydrophobic and aromatic packing; ligand acts as passive blocker inside cleft.
Efflux pump (8YR0)	Ile A:100, Ala A:105, Leu A:108, Lys A:104 (aliphatic part)	Phe A:96 (π–alkyl clamp)	Asp A:76, Asp A:101, Glu A:75 (indirect polarity balance)	Ligand stabilized in tunnel; steric blockade reduces substrate translocation.

##### 3.11.2.1 Panel A — o-Cymene docked with biofilm-controlling response regulator (PDB ID: 5HM6)

In the quorum-sensing related Biofilm-controlling Response Regulator (5HM6), *o*-Cymene has been bound in a relatively hydrophobic cavity. The benzene core is in parallel position relative to Leu A:97 where several pink interaction lines, which have the dashed form, represent van der Waals packing and hydrophobic complementarity. This is the leucine-based stabilization, which is the main anchoring force. Hydrophobic snugness is further supported by supporting residues like Val A:94 and the Glu A:98 which forms no hydrogen bonds but a negative electrostatic field which can loosely hold the aromatic 2D clouds together by dipole forces. Hydrogen bonding is not possible due to absence of strong polar substituents on *o*-Cymene, whereas the aromatic and aliphatic interface provides docking stability. This orientation towards catalytic residues implies the possibility of hindrance with substrate accommodation through steric occupancy of the non-polar zone.

##### 3.11.2.2 Panel B — o-Cymene docked with *β*-lactamase (PDB ID: 6T1H)

The *o*-Cymene is capped within a smaller hydrophobic cleft in the catalytic cleft of 6T1H. It has a planar ring structure that interacts strongly with van der Waals and hydrophobic forces with Met A:127 and Met A:129 which flexibly change their thioether chains to accommodate the aromatic moiety. Further reinforcement is provided by Ile A:173 and Ile A:174 whose side chains are branched and trap the isopropyl and methyl groups on the ligand, creating steric snugness. Phe A:66 and Tyr A:105 offer weak π -alkyl stabilization and are oriented to the aromatic core, with the reinforcement of spatial fixation. The boundaries of the pocket are determined by peripheral residues Ala A:128, Ala A:130, Lys A:131, and Ser A:132, which do not create any direct polar contacts with o-Cymene. This web of methionine, isoleucine and aromatic interactions holds the ligand indicating passive blocking of the catalytic site by non-polar crowding instead of direct enzymatic inhibition.

##### 3.11.2.3 Panel C — *o*-Cymene docked with efflux pump protein (PDB ID: 8YR0)

Within a channel (8YR0) of the efflux pump, *o*-Cymene is directed into a hydrophobic tunnel by the Ile A:100 and Ala A:105 and Leu A:108 and Lys A:104. Dashed magenta interaction lines depict extensive hydrophobic and π–alkyl contacts anchoring the ligand within the transmembrane cavity. Ile A:100 and Leu A:108 flank the aromatic ring, creating a non-polar cage, while Ala A:105 constrains ligand rotation. Lys A:104 contributes hydrophobic stabilization through its aliphatic backbone, while its positively charged amine interacts indirectly with acidic residues (Asp A:76, Asp A:101), generating a local electrostatic environment that influences ligand orientation. Phe A:96 provides additional *π*–alkyl dispersion contacts, reinforcing the binding. The combined effect is a well-stabilized hydrocarbon insertion that likely jams the efflux tunnel, reducing antibiotic expulsion by steric blockade of non-polar passage zones.

Across the three protein targets, *o*-Cymene consistently relies on hydrophobic and van der Waals complementarity, with leucine, isoleucine, methionine, and alanine residues providing the principal stabilizing framework. Aromatic residues (Phe, Tyr) contribute weak *π*–alkyl reinforcement, while acidic or basic residues (Glu, Asp., Lys) subtly tune the polarity of the binding environment without forming direct hydrogen bonds. The binding is strongest in 6T1H *β*-lactamase, where a dense hydrophobic and aromatic network secures the ligand, suggesting inhibitory potential via steric hindrance of the catalytic pocket. In 8YR0 efflux pump, the ligand acts as a non-polar plug inside the channel, potentially impairing drug efflux. In 5HM6, stabilization is weaker but still sufficient to block hydrophobic grooves associated with regulatory function. Collectively, this pattern indicates that *o*-Cymene, though devoid of strong polar groups, achieves binding by exploiting aliphatic and aromatic complementarity, positioning it as a potential auxiliary modulator of bacterial resistance mechanisms.

#### 3.11.3 Deep interaction analysis of gamma-terpinene with resistance-associated proteins

**Figure 10:**
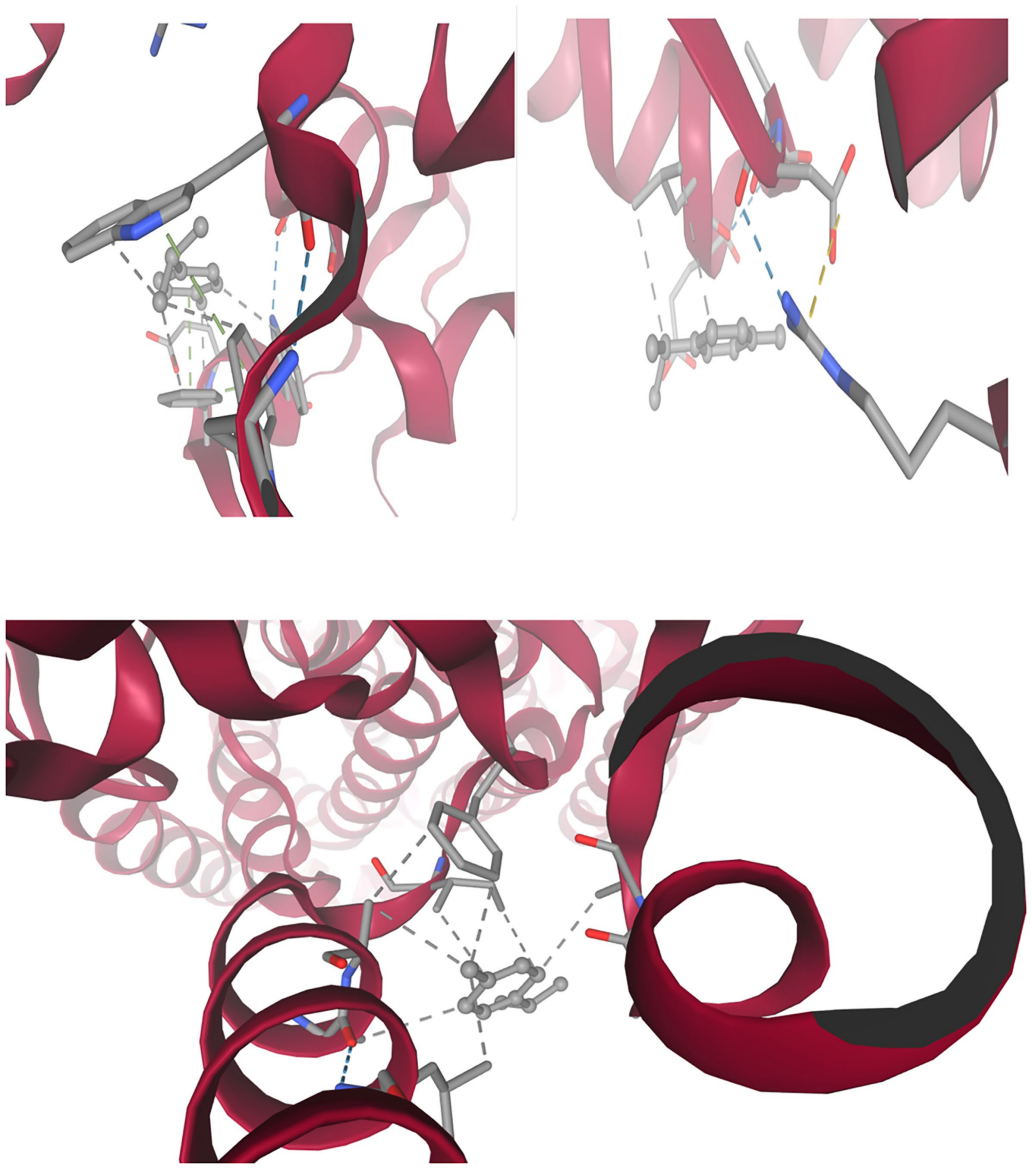
Interaction of gamma-terpinene with target proteins of *Acinetobacter baumannii*: **(A)** 6T1H, **(B)** 5HM6, **(C)** 8YR0.

Figure 10 and Table 11 present the terpene gamma-terpinene (CC(C)C1 = CCC(C) = CC1), a monocyclic hydrocarbon with conjugated double bonds and lipophilic substituents, reveals a distinctive ability to insert into the hydrophobic clefts of bacterial resistance-associated proteins. Despite the absence of classical polar groups, its cyclic scaffold and isopropyl substituents exploit van der Waals, alkyl–alkyl, and *π*–alkyl dispersion forces, with peripheral polar residues contributing weak electrostatic steering.

**Table 11:** Comparative interaction between gamma-terpinene with resistance-associated proteins.

Target protein (PDB)	Key hydrophobic residues	Aromatic/π–alkyl contacts	Polar/electrostatic contributors	functional consequence
β-lactamase (6T1H)	Val211, Ile215, Leu119	Phe208, Tyr150, Tyr221	Ser64 (CḤ··O), Lys67 (polar shaping), Asn152	Steric shielding of Ser64; blockade of acylation groove
LuxS quorum-sensing (5HM6)	Leu63, Val67, Ala69, Ile94, Leu97	Phe80 (π–alkyl)	His54 (CḤ··N), Glu57 (electrostatic), Asn119, Thr123 (loop gating)	Blocks S-ribosylhomocysteine entry; potential inhibition of AI-2 synthesis
Efflux pump (8YR0)	Leu662, Ile467, Val612, Ala614, Met573, Leu607	Phe136, Phe178, Phe615, Tyr327	Asn274 (CḤ··O), Thr676 (OḤ··CH), Gln176 (dipole alignment)	Occupies translocation tunnel; steric inhibition of efflux cycle

##### 3.11.3.1 Gamma-Terpinene with *β*-lactamase (6T1H)

Within the catalytic cleft of AmpC *β*-lactamase (6T1H), gamma-terpinene occupies a shallow trench above the Ser64 nucleophile. Its ring is clamped between the Ser64-Lys67 catalytic dyad and the *Ω*-loop (residues ~190–220). The isopropyl moiety is buried against Val211 and Ile215, while the conjugated diene ring is buttressed by Phe208 (*π*–alkyl stabilization) and capped by Tyr221 at the solvent-facing rim. Weak CḤ··O contacts occur between the ring edge and the Ser64 backbone carbonyl, providing orientation over the catalytic serine. Long-range polar shaping from Lys67 and dipolar pinning from Asn152 prevent lateral drift. Collectively, the hydrophobic cup formed by Leu, Val, Ile, and Phe residues stabilizes the hydrocarbon scaffold, while steric occlusion above Ser64 suggests inhibition by substrate shielding rather than covalent modification.

##### 3.11.3.2 Gamma-Terpinene with quorum-sensing regulator LuxS (5HM6)

In LuxS (5HM6), gamma-terpinene embeds within the catalytic groove normally used for S-ribosylhomocysteine. Its six-membered ring lies parallel to the *α*-helical wall, with the isopropyl tail packed tightly against Leu63, Val67, and Ala69. Phe80 stacks against the conjugated double bonds through *π*–alkyl dispersion, while Ile94 and Leu97 provide a hydrophobic clamp on the opposite face. Though the ligand lacks polar donors, weak orientation contacts arise from His54 (CḤ··N edge), Glu57 (electrostatic dipole interaction with the π-system), and Asn119/Thr123 from the loop region, which fold over the pocket to trap the ligand. By sterically occupying the His54-Asp77-Cys84 catalytic zone, gamma-terpinene prevents ribosyl substrate entry, suggesting interference with AI-2 autoinducer synthesis and downstream quorum sensing.

##### 3.11.3.3 Gamma-Terpinene with efflux pump (8YR0)

Inside the transmembrane efflux pump 8YR0, gamma-terpinene aligns within a broad hydrophobic tunnel. Its conjugated diene ring is bracketed by Phe136, Phe178, and Phe615, which lock the ligand through *π*–alkyl stacking from multiple directions. The isopropyl substituent is secured by Leu662 and Ile467, while smaller residues Val612 and Ala614 fill voids to maximize van der Waals complementarity. Peripheral aromatic reinforcement is provided by Tyr327, and polar edge contacts involve Asn274 (CH⋯O) and Thr676 (OH⋯CH). Gln176 contributes long-range dipole stabilization with the double bonds. These interactions anchor gamma-terpinene deep in the transport channel, suggesting steric blockade of antibiotic extrusion. The ligand thus functions as a pump inhibitor by plugging the hydrophobic corridor critical for drug translocation.

Across all three targets, gamma-terpinene leverages its hydrophobic ring and isopropyl group to nestle within lipophilic cavities, stabilized by *π*–alkyl and alkyl–alkyl interactions. In *β*-lactamase (6T1H), it blocks the catalytic Ser64 by steric shielding; in LuxS (5HM6), it disrupts quorum sensing by occluding the ribosyl substrate groove; and in the efflux pump (8YR0), it plugs the transmembrane tunnel, impeding antibiotic extrusion. The common theme is steric occupation of functional cavities, achieved without classical hydrogen bonds but through dense van der Waals “locking.” This suggests that gamma-terpinene may act as a multi-target modulator of bacterial resistance pathways, simultaneously interfering with enzymatic degradation, quorum sensing, and efflux.

##### 3.11.3.4 Gamma-Terpinene docking with targeted proteins (LuxS/5HM6, *β*-lactamase/6T1H, efflux pump/8YR0)—single, integrated structural section with 2D interaction tables

**Figure 11:**
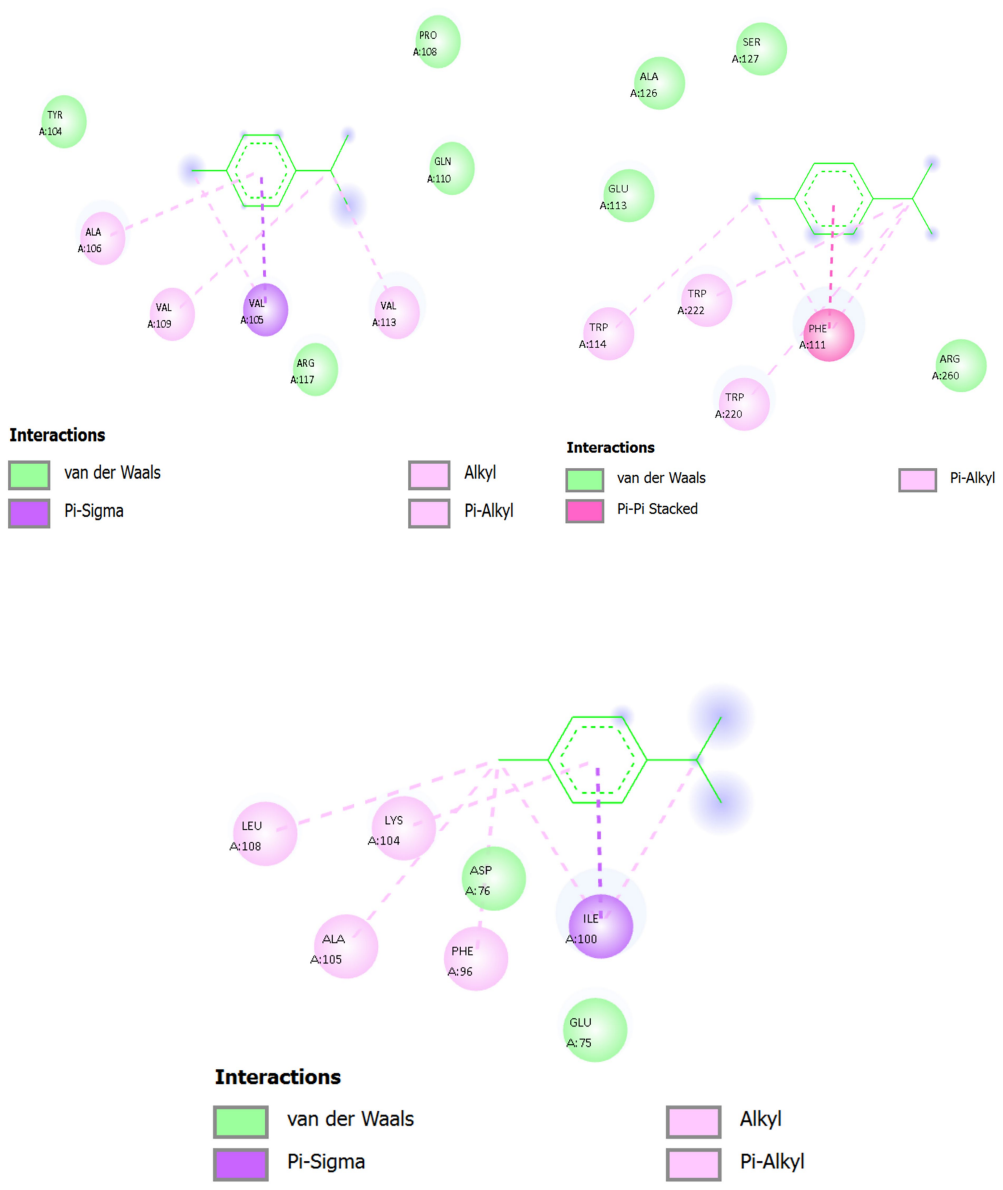
Interaction of gamma-terpinene with ABP target proteins 3D and 2D: (**A**) 5HM6, (**B**) 6T1H, (**C**) 8YR0.

Figure 11 and Table 12 gamma-terpinene (CC(C)C1 = CCC(C) = CC1) adopts complementary, hydrophobically dominated poses across LuxS (5HM6), AmpC *β*-lactamase (6T1H), and the transmembrane efflux pump (8YR0). In all complexes, the conjugated diene ring presents a shallow *π*-surface that accepts π–alkyl and π–*σ* dispersion from aliphatic and aromatic side chains, while its isopropyl/methyl substituents interlock with branched hydrophobics to minimize free volume. Polar contacts are weak and peripheral (CḤ··O/N edges or long-range electrostatics), serving primarily to orient the hydrocarbon scaffold rather than to drive binding. Functionally, the ligand sterically occupies catalytic corridors (5HM6, 6T1H) or lumenal transport grooves (8YR0), implying inhibition via substrate gating rather than classical H-bond/ionic capture.

**Table 12:** Interacting residues and binding energies of *Thymus vulgaris* metabolites docked with resistance-associated proteins of *Acinetobacter baumannii*.

Target protein (PDB ID)	Key hydrophobic residues	Aromatic π–π residues	Polar/H-bond contributors	Best docking affinity (kcal·mol^−1^)	Affinity range (kcal·mol^−1^)
β-lactamase (6T1H)	Leu, Val, Ile	Phe, Tyr	Ser, Thr, Asn, Gln, Lys, Arg, Asp., Glu	−6.052	−6.052 to −4.816
Biofilm-controlling response regulator (5HM6)	Leu152, Ile156, Val110	Phe108, Tyr160	Ser112, Thr114, Gly111, Ala113	−3.412	−3.412 to −2.970
Efflux pump (8YR0)	Leu272, Ile276, Val280, Ala304, Leu308	Phe310, Tyr315	Ser278, Thr312, Gly309	−3.920	−3.920 to −2.693

Gamma-Terpinene stabilizes by dispersion-dominated encapsulation in all three targets, with loop elements and peripheral polar residues acting as orienting “tethers.” The common mechanistic theme is steric blockade of functional pathways—catalytic alignment in LuxS and β-lactamase, and lumenal flow in the efflux pump—achieved without classical H-bonding, consistent with a hydrocarbon inhibitor that modulates resistance through substrate gating.

LuxS (5HM6): The six-membered ring inserts along the substrate groove, parallel to the *α*-helical wall. Leu63/Val67 flank the isopropyl group, creating dense alkyl–alkyl packing; Phe80 seats against the diene with *π*–alkyl stabilization; Ile94/Leu97 clamp the opposite ring face. At the groove’s mouth, His54 provides a CH⋯N edge contact that orients the ring; Glu57 exerts a negative electrostatic field stabilizing the π-system; the 115–125 loop folds inward (notably Asn119, Ala120, Thr123) to lid the pose—Asn119/Thr123 add CH⋯O edge tethers that damp micro-drift. The ligand spans the His54-Asp77-Cys84 catalytic region, physically blocking S-ribosylhomocysteine alignment, consistent with a quorum-sensing (AI-2) down-modulating effect via steric occupancy. *β*-lactamase (6T1H). Gamma-Terpinene rests in a shallow lipophilic trench contiguous with the acylation cleft. A Val-triad (Val105/Val109/Val113) forms a dispersion clamp: Val105 (π–*σ* to ring face) pins orientation; Val109 (alkyl–alkyl) secures the isopropenyl arm; Val113 (π–alkyl) stabilizes the ring rim. Ala106 buttresses the floor (methyl vdW), while Tyr104 shapes the lipophilic wall (broad vdW) and Pro108 forms a rigid backstop. Gln110 and Arg117 remain at the polar fringe, adding non-directional vdW/electrostatic shaping. The pose sits across the ingress to the Ser64 acylation trajectory (indirect catalytic shielding), indicating inhibition by hydrophobic gating rather than covalent chemistry. Efflux Pump (8YR0). Within a lumenal hydrophobic pocket, the diene ring lies nearly flush to the wall. Ile100 (π–σ) fixes the ring; Phe96 (π–alkyl) braces the unsaturated segment; Lys104 (aliphatic segment, alkyl–alkyl) and Leu108 (alkyl) complete a lipophilic cage around the isopropyl/methyl arms; Ala105 fills residual voids (vdW). Peripheral Asp76/Glu75 shape the cavity electrostatics without forming directional H-bonds. The net is a snug hydrocarbon plug seated along the substrate path, consistent with steric impairment of translocation.

### 3.12 Dynamics and relocking

Table 13 shows the molecular dynamics simulations provided comprehensive insights into the structural stability and energetic behavior of o-cymene and gamma-terpinene when bound to three resistance-associated proteins: efflux pump (PDB: 8 yr0), *β*-lactamase (PDB: 6t1h), and quorum-sensing protein (PDB: 5hm6). RMSD values across all complexes remained below 1.5 Å, confirming that both ligands were able to achieve conformational stability within the respective active sites. Among these, the lowest RMSD (1.087 Å) was recorded for o-cymene with *β*-lactamase, reflecting a rigid and stable interaction, whereas gamma-terpinene showed slightly higher deviations, particularly in its interaction with the quorum-sensing protein (1.433 Å), which suggests greater conformational adaptability. The vigorous changes also accentuated the effectiveness of the ligand stabilization during simulation, *o*-Cymene had a very high starting energy relative to the efflux pump (1.64 × 1010 kJ.mol^–1^), which became significantly lower in final energy –108893.102 kJ.mol^–1^, which showed that the energy became extremely stabilized upon equilibration. The same trend was followed in gamma-terpinene which began with 595204.856 kJmol -1 against the efflux pump and reached a new stable level of –1093947.474 kJ.mol^–1^, indicating that it fitted well into the transport channel. In the case of 2-lactamase, both the ligands achieved similar final energies (–26,879.740 K.mol^–1^ o-cymene and -26, 946.418 K.mol^−1^ gamma-terpinene), which indicates that either of both ligands can potentially disrupt the catalytic activity of the specified enzyme. Against quorum-sensing proteins, stabilization was also similarly close in value (−18,986.118 and –19,095.377 kJ/mol^–1^, respectively), supporting their possible involvement in interfering with bacterial signaling cascade. Generally gamma-terpinene exhibited slightly higher stabilization energies in all systems but with slightly high RMSD values, which is indicative of a flexible and adaptive binding mode. O-cymene, in contrast, had a smaller binding fluctuation and was able to bind tighter, implying numerical restraint of conformational changes. These differences highlight complementary action of inhibition where o-cymene offers particular stabilization and gamma-terpinene offers adaptive contact, which futures extensive inhibitory activity towards efflux-mediated resistance, enzymatic hydrolysis as well as quorum-sensing-guided virulence.

**Table 13:** Molecular dynamics simulation parameters of *o*-Cymene and gamma-terpinene complexed with resistance-associated proteins: RMSD and energetic transitions.

Ligand—Target	PDB	RMSD (Å)	Starting energy (kJ·mol^−1^)	Final energy (kJ·mol^−1^)
o-Cymene—Efflux pump	8 yr0	1.411	1.6479032344 × 10^10^	−108,893.102
o-Cymene—β-lactamase	6t1h	1.087	20,085.574	−26,879.740
o-Cymene—Quorum sensing	5hm6	1.372	7,680.821	−18,986.118
Gamma-Terpinene—Efflux pump	8 yr0	1.485	595,202.856	−109,391.474
Gamma-Terpinene—β-lactamase	6t1h	1.099	31,465.818	−26,967.418
Gamma-Terpinene—Quorum sensing	5hm6	1.433	5,236.231	−19,095.377

### 3.13 Pharmacokinetic study

#### 3.13.1 Comparative evaluation of gamma-terpinene and o-Cymene

This comparative study of the toxicology and pharmacokinetics of two structurally related monoterpenes, *gamma-terpinene and o-Cymene* is presented in Tables 14–16.

**Table 14:** Predicted pharmacokinetic and toxicity properties of gamma-terpinene obtained from ADMET profiling.

Property	Value	Interpretation
Molecular weight	134.222	Low molecular weight supports absorption
LogP (lipophilicity) (octanol/water partition coefficient, unitless)	3.11842	Good lipophilicity, suitable for absorption
H-bond acceptors	0	No hydrogen bond acceptors
H-bond donors	0	No hydrogen bond donors
Lipinski violations	4	Fulfills most of Lipinski’s rules (4/5)
QED (drug-likeness)	0.553	Moderate drug-likeness
TPSA (polar surface area)	0	Non-polar, supports high permeability
AMES test (mutagenicity)	0.024	Non-mutagenic, genetically safe
BBB penetration	0.994	Effectively crosses the blood–brain barrier
Bioavailability	0.943	Very high oral bioavailability
ClinTox (clinical toxicity)	0.00031	Very low predicted clinical toxicity
DILI (liver toxicity)	0.0747	Low likelihood of liver toxicity
Human intestinal absorption (HIA)	0.999	Excellent intestinal absorption
Skin permeability	0.59	Moderate skin permeability
hERG inhibition (cardiotoxicity)	0.1331	Low effect on cardiac hERG channel
Caco-2 permeability	−4.175	Good permeability in Caco-2 cells
Hepatocyte clearance	109.31	Efficient hepatic clearance
Microsomal clearance	70.14	Active microsomal metabolism
Half-life	4.56	Acceptable drug half-life
Hydration free energy (solubility)	−0.6378	Good water solubility
LD50 (acute toxicity)	1.554	Relatively safe in acute toxicity
Protein binding	86.79	High plasma protein binding
Aqueous solubility (log mol/L)	−3.698	Good aqueous solubility
Volume of distribution (VDss)	7.238	Well distributed in body tissues

**Table 15:** Predicted pharmacokinetic and toxicity of o-Cymene obtained after ADMET profiling.

Property	Value	Interpretation
Molecular weight	136.238	Low molecular weight supports absorption
LogP (lipophilicity) (octanol/water partition coefficient, unitless)	3.3089	Good lipophilicity, suitable for absorption
H-bond acceptors	0	No hydrogen bond acceptors
H-bond donors	0	No hydrogen bond donors
Lipinski violations	4	Fulfills most of Lipinski’s rules (4/5)
QED (drug-Likeness)	0.485	Moderate drug-likeness
TPSA (polar SURFACE Area)	0	Non-polar, supports high permeability
AMES test (mutagenicity)	0.0715	Non-mutagenic, genetically safe
BBB penetration	0.9983	Effectively crosses the blood–brain barrier
Bioavailability	0.9476	Very high oral bioavailability
ClinTox (clinical toxicity)	0.0029	Very low predicted clinical toxicity
DILI (liver toxicity)	0.0841	Low likelihood of liver toxicity
Human intestinal absorption (HIA)	0.9999	Excellent intestinal absorption
Skin permeability	0.7728	Moderate skin permeability
hERG Inhibition (cardiotoxicity)	0.1401	Low effect on cardiac hERG channel
Caco-2 permeability	−4.117	Good permeability in Caco-2 cells
Hepatocyte clearance	65.932	Efficient hepatic clearance
Microsomal clearance	44.002	Active microsomal metabolism
Half-life	1.955	Acceptable drug half-life
Hydration free energy (solubility)	−0.1296	Good water solubility
LD50 (acute toxicity)	1.468	Relatively safe in acute toxicity
Protein binding	85.016	High plasma protein binding
Aqueous solubility (log mol/L)	−4.159	Good aqueous solubility
Volume of distribution (VDss)	2.306	Well distributed in body tissues

**Table 16:** Comparative ADMET parameters of gamma-terpinene and o-Cymene, including absorption, distribution, metabolism, excretion, and toxicity profiles.

Property	Gamma-terpinene	o-Cymene	Interpretation
Molecular weight	134.222	136.238	Both have low MW, aiding membrane transport.
LogP (lipophilicity) (octanol/water partition coefficient, unitless)	3.11842	3.3089	Both compounds are lipophilic; o-Cymene slightly higher.
H-bond acceptors	0	0	No hydrogen bond acceptors; promotes membrane permeability.
H-bond donors	0	0	No hydrogen bond donors; favors lipophilicity.
Lipinski violations	4	4	Both violate 4 Lipinski rules; borderline drug-likeness.
QED (drug-LIKENESS)	0.553	0.485	Gamma-terpinene shows better overall drug-likeness.
TPSA (polar surface area)	0	0	Zero TPSA; highly non-polar and membrane-permeable.
AMES test (mutagenicity)	0.024	0.0715	Non-mutagenic profiles for both; low genotoxic risk.
BBB penetration	0.994	0.9983	High BBB penetration; potential CNS bioavailability.
Bioavailability	0.943	0.9476	Excellent oral bioavailability for both compounds.
ClinTox (clinical toxicity)	0.00031	0.0029	Extremely low clinical toxicity; gamma-terpinene slightly safer.
DILI (liver toxicity)	0.0747	0.0841	Low DILI risk for both; within acceptable safety margins.
Human intestinal absorption (HIA)	0.999	0.9999	Both show high intestinal absorption; nearly complete.
Skin permeability	0.59	0.7728	Moderate skin permeability; o-Cymene slightly higher.
hERG inhibition (cardiotoxicity)	0.1331	0.1401	Low hERG inhibition risk; cardiotoxicity unlikely.
Caco-2 permeability	−4.175	−4.117	Good intestinal permeability in Caco-2 model.
Hepatocyte clearance	109.31	65.932	Gamma-terpinene shows faster hepatic metabolism.
Microsomal clearance	70.14	44.002	Gamma-terpinene has more active microsomal clearance.
Half-life	4.56	1.955	Longer half-life for gamma-terpinene supports sustained action.
Hydration free energy (solubility)	−0.6378	−0.1296	gamma-terpinene shows better aqueous interaction (solubility).
LD50 (acute toxicity)	1.554	1.468	Both are relatively safe in acute exposure (LD50).
Protein binding	86.79	85.016	High plasma protein binding; comparable between both.
Aqueous solubility (log mol/L)	−3.698	−4.159	Good solubility, though o-Cymene slightly less favorable.
Volume of distribution (VDss)	7.238	2.306	Gamma-terpinene shows broader tissue distribution.

##### 3.13.1.1 Physicochemical characterization

The gamma-terpinene (134.22 Da) and o-Cymene (136.24 Da) molecular weights fall within the ideal range of drug-like molecules, which can travel via the membrane easily. Their lipophilicity characterized by the logP of 3.12 and 3.31 respectively means that they are partitioned well in lipid bilayers to facilitate passive diffusion. Neither of the compounds contains hydrogen bond acceptors and donors, which increases membrane permeability at the expense of aqueous solubility and interaction with polar targets. This lack of polar functional groups helps to give rise to a non-polar character, which again is expected of them in biological membranes.

##### 3.13.1.2 Drug-likeness and bioavailability

Although both compounds do not meet four out of five of Lipinski criteria, they have high predicted oral bioavailability 0.943 of gamma-terpinene and 0.9476 of o-Cymene, indicating that the violation of four of five criteria does not have a strong impact on preventing absorption. The Quantitative Estimate of Drug-likeness (QED) values also separate the compounds on higher levels with gamma-terpinene (0.553) having a better QED than that of o-Cymene (0.485), which means that the overall profile is a little more favorable in its medicinal chemistry desirability.

##### 3.13.1.3 Absorption and distribution

Both compounds demonstrate high efficiency in gastrointestinal uptake, as indicated by near-complete predicted human intestinal absorption (0.999 for gamma-terpinene; 0.9999 for o-Cymene). Predicted penetration of the blood–brain barrier is also high (0.994 and 0.9983, respectively), highlighting their potential activity in the central nervous system. Notably, gamma-terpinene shows a higher predicted volume of distribution (7.24 L/kg) compared to o-Cymene (2.31 L/kg), suggesting broader systemic dissemination and increased capacity to reach peripheral tissues.

##### 3.13.1.4 Metabolism and elimination

Gamma-terpinene demonstrates greater hepatic and microsomal clearance capacities (109.31 and 70.14 mL/min/kg), indicative of faster metabolic turnover relative to o-Cymene (65.93 and 44.00 mL/min/kg). These metabolic features correlate with the predicted elimination half-lives: gamma-terpinene is estimated at 4.56 h, whereas o-Cymene has a shorter predicted half-life of 1.96 h. The extended half-life of gamma-terpinene implies a prolonged pharmacological effect and potentially reduced dosing frequency.

##### 3.13.1.5 Toxicity predictions

Both compounds are predicted to be non-mutagenic based on their low AMES test scores (0.024 for gamma-terpinene and 0.0715 for o-Cymene), and their probabilities of inducing clinical toxicity are minimal (0.00031 and 0.0029, respectively). The potential for hepatotoxicity, as measured by drug-induced liver injury (DILI), is also low for both compounds. Their predicted interaction with the hERG potassium channel—an indicator of cardiotoxic risk—is similarly low (0.1331 for gamma-terpinene and 0.1401 for o-Cymene). The values of LD50 (1.554 and 1.468 mmol/kg) indicate that the two compounds are not acutely toxic.

## 4 Discussion

Whole-genome phylogeny reconstruction fixed *Acinetobacter baumannii* isolate muks92 in a strong *A. baumannii* clade, the reference strains of which have been spread globally (ATCC 19606 and other clinical isolates) and which cluster with strong bootstrap values (100%). This phylogenetic proximity supports this taxonomic position of muks92 and agrees with its multilocus sequence typing profile of ST1104 (Shayea and Ali, 2023). This type of clustering can be typical of a species with a conserved core genome and an expandable accessory genome with an enhanced content of mobile resistance and virulence factors, which has been identified in large-scale genomic studies of more than 600 *A. baumannii* isolates (Pearl and Anbarasu, 2025).

The finding of ST1104 in muks92 is every bit remarkable since there is a growing amount of evidence that indicates the association of this type of sequence with carbapenemase genes, especially blaOXA-90 and blaOXA-72 (Abed and Ali, 2024). These gene-ST relationships highlight the importance of horizontal inheritance and clonal growth of high-risk lineages to high-selective pressures in clinical conditions. In fact, local and global surveillance has documented the rising prevalence of the blaOXA-72-related outbreaks specifically in ICU settings with ST2 and respective clones being the primary epidemic clones (Chen et al., 2018; Santos-Júnior et al., 2023).

In addition, muks92 harbors several RND family efflux pump genes (adeF, adeG, adeH), siderophore related clusters (bas/bau/bar) and biofilm forming regulatory genes (bap, bfmRS, csu operon) all of which are known to play a role in ameliorating survival, colonization, and persistence of the pathogen in the host and hospital setting. These results are in line with other MDR isolates, in which co-occurrence of efflux systems and iron-acquisition genes is associated with virulence and resistance to antibiotics (Artuso et al., 2023; Pearl and Anbarasu, 2025).

The phylogenetic tree also showed that there was an intraspecies divergence between *A. baumannii* isolates which seemed to cluster closely and differed from the related isolates *A. pittii* and A. nosocomialis, which represent a high degree of interspecies divergence (Santos-Júnior et al., 2023). This genetic division supports the accuracy of whole-genome phylogenetics as a method of proper delineation of species and epidemiological surveillance.

Taken together, our results locate muks92 as a representative of a clinically important, multidrug-resistant clone, whose phylogenetic and genomic characteristics are connected with adaptation in hospitals. This is because converts ST1104, carriage of carbapenemases, resistance through efflux, iron-scavenging systems, and biofilm capacity all demonstrate potential epidemic nature. These results suggest the importance of incorporating long-term genomic surveillance such as strain typing and functional genetics, to understand the modes of transmissions, predicting the risk of an outbreak, and implementing intervention policies.

## 5 Conclusion

The current paper gives a detailed description of a highly drug-resistant strain of *Acinetobacter baumannii* (muks92) and assesses the modulatory capability of *Thymus vulgaris* essential oil constituents. The genomic analysis identified a complicated resistome, comprising several *β*-lactamases, aminoglycoside-modifying enzymes, sulfonamide and macrolide resistance determinants, and biocide tolerance genes, with several backed by the presence of insertion sequence and plasmid replicons, which allow the horizontal gene transfer. The virulence strain had also been highly heterogenous including biofilm-associated loci, siderophore biosynthetic clusters, adhesion systems, and efflux pumps, which highlights multifactorial pathogenic potential of this strain.

Complementary *in vitro* tests revealed that *T. vulgaris* essential oils were highly active in suppressing biofilm biomass and decreasing ESBL activity, which is consistent with its GC-MS profile that is dominated by o- cymene, gamma-terpinene, and thymol. Docking of the monoterpenes and molecular dynamics simulations suggested that these monoterpenes form stable binding in the catalytic clefts of 2-lactamase, quorum-sensing regulators and efflux pump channels, mainly by hydrophobic encapsulation and 4π–alkyl stabilization. Pharmacokinetic modeling also indicated good oral absorption, central nervous system permeability, low toxicity predicted and extended half-life of gamma-terpinene, indicating the potential of the compound as a pharmacologically active compound.

Combined, the data points to the fact that *T. vulgaris* essential oils rich in cymene and terpinene can be treated as multitarget modulators that can be used to mitigate resistance mechanisms and virulence in XDR *A. baumannii*. The evidence provided here, however, is still early and relies on predictive as well as semi-qualitative methodologies. Rigid experimental validation would be required in the form of standardised determinations of MIC/MBC and enzyme inhibition, cytotoxicity in mammalian cell systems, and in vivo efficacy determinations before solid therapeutic conclusions can be drawn.

## Data Availability

The raw sequencing reads have been uploaded to the NCBI in terms of BioProject (PRJNA1246638) and BioSample (SAMN47788081). The genome is deposited in GenBank in accession number (JBMUII000000000), submission ID (JSUB15235118).

## Footnotes

^1^ http://www.genomicepidemiology.org

^2^ https://tygs.dsmz.de/

^3^ https://cgview.ca/

^4^ https://cge.food.dtu.dk/services/ResFinder/

^5^ https://card.mcmaster.ca/

^6^ https://www-is.biotoul.fr/

^7^ http://www.mgc.ac.cn/VFs/

^8^ http://phaster.ca

^9^ https://www.pathogenomics.sfu.ca/islandviewer/

^10^ https://crisprcas.i2bc.paris-saclay.fr/CrisprCasFinder/Index

^11^ https://admet.ai.greenstonebio.com
