# Supplementary material for: Integrated in silico–in vitro and pharmacokinetic profiling of Thymus vulgaris-derived metabolites targeting multidrug resistance pathways in extensively drug-resistant Acinetobacter baumannii (muks92)
Source: Front Microbiol. 2025 Dec 17;16:1680686. doi: 10.3389/fmicb.2025.1680686 (PMC12753994; doi:10.3389/fmicb.2025.1680686)
Supplement: Supplementary file 1 [file Table_1.docx]

Supplementary 1 : GC_MS of THYMUS VULGARIS OIL


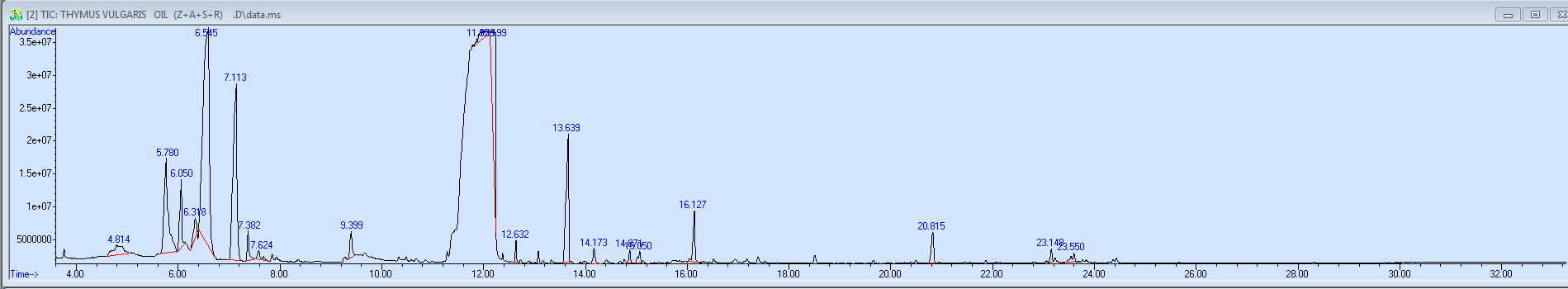


Data Path : D:\MassHunter\GCMS\1\data\

Data File : THYMUS VULGARIS OIL (Z+A+S+R) .D

Acq On : 22 Jan 2023 12:32

Operator :

Sample : THYMUS VULGARIS OIL (Z+A+S+R)

Misc :

ALS Vial : 3 Sample Multiplier: 1 زعتر

Search Libraries: C:\GCMS\firmware\NIST11.L Minimum Quality: 70

Unknown Spectrum: Apex

Integration Events: RTE Integrator - rteint.p

Pk# RT Area% Library/ID Ref# CAS# Qual

_____________________________________________________________________________

1 4.816 2.37 C:\GCMS\firmware\NIST11.L

(1R)-2,6,6-Trimethylbicyclo[3.1.1] 15854 007785-70-8 52

hept-2-ene

.alpha.-Pinene 15699 000080-56-8 64

Bicyclo[3.1.0]hex-2-ene, 2-methyl- 15898 002867-05-2 80

5-(1-methylethyl)-

2 5.777 9.92 C:\GCMS\firmware\NIST11.L

1-Nonen-3-ol 19945 021964-44-3 42

1-Octen-3-ol 12448 003391-86-4 58

1-Nonen-3-ol 19943 021964-44-3 38

3 6.054 3.51 C:\GCMS\firmware\NIST11.L

3-Octanol 13627 000589-98-0 40

3-Octanol 13641 000589-98-0 45

3-Octanol 13639 000589-98-0 74

4 6.322 1.48 C:\GCMS\firmware\NIST11.L

1,3-Cyclohexadiene, 1-methyl-4-(1- 15872 000099-86-5 95

methylethyl)-

1,3-Cyclohexadiene, 1-methyl-4-(1- 15877 000099-86-5 95

methylethyl)-

2-Carene 15666 000554-61-0 93

5 6.547 32.95 C:\GCMS\firmware\NIST11.L

o-Cymene 14806 000527-84-4 59

Benzene, 1,2,4,5-tetramethyl- 14872 000095-93-2 80

Benzene, 1,2,3,5-tetramethyl- 14870 000527-53-7 64

6 7.109 16.85 C:\GCMS\firmware\NIST11.L

.gamma.-Terpinene 15708 000099-85-4 94

.gamma.-Terpinene 15711 000099-85-4 80

.gamma.-Terpinene 15709 000099-85-4 86

7 7.378 1.47 C:\GCMS\firmware\NIST11.L

p-Menth-8-en-1-ol, stereoisomer 26767 007299-40-3 94

Cyclohexanol, 1-methyl-4-(1-methyl 26903 007299-41-4 93

ethenyl)-, cis-

2-Cyclohexen-1-ol, 1-methyl-4-(1-m 26929 029803-81-4 38

ethylethyl)-, trans-

8 7.620 0.63 C:\GCMS\firmware\NIST11.L

Cyclohexene, 1-methyl-4-(1-methyle 15858 000586-62-9 96

thylidene)-

Cyclohexene, 1-methyl-4-(1-methyle 15862 000586-62-9 93

thylidene)-

2-Carene 15677 000554-61-0 64

9 9.403 1.32 C:\GCMS\firmware\NIST11.L

3-Cyclohexen-1-ol, 4-methyl-1-(1-m 26914 020126-76-5 94

ethylethyl)-, (R)-

Terpinen-4-ol 26662 000562-74-3 93

Terpinen-4-ol 26655 000562-74-3 89

10 11.956 1.56 C:\GCMS\firmware\NIST11.L

Phenol, 2,3,5,6-tetramethyl- 23753 000527-35-5 90

Ethanone, 1-(2-hydroxy-5-methylphe 24528 001450-72-2 59

nyl)-

Ethanone, 1-(2-hydroxy-5-methylphe 24526 001450-72-2 64

nyl)-

11 12.199 11.30 C:\GCMS\firmware\NIST11.L

Phenol, 2,3,5,6-tetramethyl- 23753 000527-35-5 87

Ethanone, 1-(2-hydroxy-5-methylphe 24526 001450-72-2 64

nyl)-

Phenol, 2,3,4,6-tetramethyl- 23750 003238-38-8 43

12 12.632 0.72 C:\GCMS\firmware\NIST11.L

3-Allyl-6-methoxyphenol 33304 000501-19-9 98

Eugenol 33239 000097-53-0 96

Eugenol 33238 000097-53-0 98

13 13.636 8.18 C:\GCMS\firmware\NIST11.L

Caryophyllene 64275 000087-44-5 94

Caryophyllene 64272 000087-44-5 99

Bicyclo[5.2.0]nonane, 2-methylene- 64421 242794-76-9 68

4,8,8-trimethyl-4-vinyl-

14 14.172 0.81 C:\GCMS\firmware\NIST11.L

1,4,7,-Cycloundecatriene, 1,5,9,9- 64408 1000062-61-9 98

tetramethyl-, Z,Z,Z-

Humulene 64257 006753-98-6 96

Humulene 64256 006753-98-6 74

15 14.873 0.53 C:\GCMS\firmware\NIST11.L

.beta.-Bisabolene 64331 000495-61-4 70

.beta.-Bisabolene 64317 000495-61-4 93

.beta.-Myrcene 15697 000123-35-3 38

16 15.046 0.66 C:\GCMS\firmware\NIST11.L

Naphthalene, 1,2,3,5,6,8a-hexahydr 64486 000483-76-1 89

o-4,7-dimethyl-1-(1-methylethyl)-,

(1S-cis)-

Naphthalene, 1,2,3,5,6,8a-hexahydr 64487 000483-76-1 97

o-4,7-dimethyl-1-(1-methylethyl)-,

(1S-cis)-

Naphthalene, 1,2,4a,5,8,8a-hexahyd 64533 000523-47-7 80

ro-4,7-dimethyl-1-(1-methylethyl)-

, [1S-(1.alpha.,4a.beta.,8a.alpha.

)]-

17 16.128 2.66 C:\GCMS\firmware\NIST11.L

Caryophyllene oxide 77539 001139-30-6 87

Caryophyllene oxide 77536 001139-30-6 91

Bicyclo[6.1.0]nonane, 9-(1-methyle 33787 056666-90-1 46

thylidene)-

18 20.811 1.75 C:\GCMS\firmware\NIST11.L

3,4-Dimethyl-o-phenylenediamine 16236 041927-01-9 10

Isobutyric acid, .alpha.-[2,4,5-tr 79074 086550-00-7 10

imethylphenyloxy]-

Allopurinol 16025 000315-30-0 10

19 23.148 0.55 C:\GCMS\firmware\NIST11.L

9-Octadecenoic acid (Z)-, methyl e 141300 000112-62-9 99

ster

cis-13-Octadecenoic acid, methyl e 141299 1000333-58-3 99

ster

9-Octadecenoic acid, methyl ester, 141310 001937-62-8 99

(E)-

20 23.546 0.79 C:\GCMS\firmware\NIST11.L

2,4-Dibromo-6-methyl-6,7-dihydro-9 171200 1000210-13-8 12

H-5-oxa-9-azabenzocyclohepten-8-on

1,5-Bis[methyl(tetramethylene)sily 144310 1000216-99-9 12

loxy]pentane

3-Buten-2-one, 1,1,1-trifluoro-4-h 73783 066929-54-2 10

ydroxy-4-phenyl-

Drug screen.M Mon Jan 23 12:03:31 2023

Supplementary 2 - Table 1 : Raw data of biofilm .

| No. | A(before treatment) | B(after treatment) | |
| --- | --- | --- | --- |
|  | 0.08 | | 0.07 |
|  | 0.07 | | 0.02 |
|  | 0.06 | | 0.05 |
|  | 0.092 | | 0.05 |
|  | 0.091 | | 0.055 |
|  | 0.082 | | 0.02 |
|  | 0.073 | | 0.02 |
|  | 0.062 | | 0.01 |
|  | 0.083 | | 0.003 |
|  | 0.073 | | 0.05 |

Supplementary 3 - Table 1 : Raw data of ESBLs .

| No. | A(before treatment) | B(after treatment) |
| --- | --- | --- |
|  | 0.32 | 0.11 |
|  | 0.72 | 0.22 |
|  | 0.5 | 0.36 |
|  | 0.7 | 0.44 |
|  | 1.2 | 0.62 |
|  | 0.67 | 0.57 |
|  | 0.9 | 0.45 |
|  | 0.7 | 0.51 |
|  | 0.7 | 0.52 |
|  | 0.334 | 0.33 |
