# Supplementary material for: Integrated in silico–in vitro and pharmacokinetic profiling of Thymus vulgaris-derived metabolites targeting multidrug resistance pathways in extensively drug-resistant Acinetobacter baumannii (muks92)
Source: Front Microbiol. 2025 Dec 17;16:1680686. doi: 10.3389/fmicb.2025.1680686 (PMC12753994; doi:10.3389/fmicb.2025.1680686)
Supplement: Supplementary file 2 [file Data_Sheet_1.docx]

**Supplementary7** **Closely related *A. baumannii* isolates (cgMLST): accession, ST, source, collection year, and AMR/virulence profiles.**

Isolate

AB5375

AB4332

VNMU_136

2MG

68SM01

72SM01

65SM01

Accession number

RJLW01

RJLV01

WYAB01

FUEX01

FUEK01

FUEW01

FUEV01

ST

944.0

944.0

944.0

944.0

944.0

944.0

944.0

Host

Human

Human

Human

–

–

–

–

Disease

Hospital- acquired infection

Hospital- acquired infection

Infection

–

–

–

–

Isolation Source

Respiratory tract

Respiratory tract

Wound

–

–

–

–

Country State

Brazil: Roraima

Brazil: Roraima

Ukraine

–

–

–

–

Collection Year

2016

16-Oct-2016

2017-08-22

–

–

–

–

Antimicrobial resistance gene

aac(3)-IIa,aadA5,ant(2″)- Ia,aph(3′)-Ia,armA,blaADC- 25,blaCTX-M-124,blaOXA- 72,blaOXA-90,blaTEM- 1B,mph(E),msr(E),sul1,sul2

aac(3)-IIa,aadA5,ant(2″)- Ia,aph(3′)-Ia,armA,blaADC- 25,blaCTX-M-124,blaOXA- 72,blaOXA-90,blaTEM- 1B,mph(E),msr(E),sul1,sul2

aac(6′)- Ian,aadA5,armA,blaADC- 25,blaCARB-14,blaCTX-M-

124,blaOXA-72,blaOXA-90,cat A1,floR,mph(E),msr(E),sul1

aac(6′)-Ian,ant(2″)-Ia,aph(3′)- Ia,blaADC-25,blaCARB- 14,blaOXA-90,floR,sul2

aac(6′)-Ian,ant(2″)-Ia,aph(3′)- Ia,blaADC-25,blaCARB- 14,blaOXA-90,floR,sul2

aac(6′)-Ian,ant(2″)-Ia,aph(3′)- Ia,blaADC-25,blaOXA- 90,floR,sul2

aac(6′)-Ian,ant(2″)-Ia,aph(3′)- Ia,blaADC-25,blaOXA- 90,floR,sul2

Virulence gene

abaR,adeF,adeG,adeH,bap,barA,barB,basA,basB,basC,basD,bas F,basG,basH,basI,basJ,bauB,bauC,bauD,bauE,bauF,bfmR,bfmS, csuA,csuA/B,csuB,csuC,csuD,csuE,entE,ompA,pgaA,pgaB,pga C,pgaD,plc,plcD

abaI,abaR,adeF,adeH,bap,barA,barB,basA,basB,basC,basD,basF

,basG,basH,basI,basJ,bauB,bauC,bauD,bauE,bauF,bfmR,bfmS,c suA,csuA/B,csuB,csuC,csuD,csuE,entE,ompA,pgaA,pgaB,pgaC

,pgaD,plc,plcD

abaI,abaR,adeF,adeG,adeH,bap,barA,barB,basA,basB,basC,bas D,basF,basG,basH,basI,basJ,bauB,bauC,bauD,bauE,bauF,bfmR, bfmS,csuA,csuA/B,csuB,csuC,csuD,csuE,entE,ompA,pgaA,pga B,pgaC,pgaD,plc,plcD

abaI,abaR,adeF,adeG,adeH,bap,barA,barB,basA,basB,basC,bas D,basF,basG,basH,basI,basJ,bauB,bauC,bauD,bauE,bauF,bfmR, bfmS,csuA,csuA/B,csuB,csuC,csuD,csuE,entE,ompA,pgaA,pga B,pgaC,pgaD,plc,plcD

abaI,abaR,adeF,adeG,adeH,bap,barA,barB,basA,basB,basC,bas D,basF,basG,basH,basI,basJ,bauB,bauC,bauD,bauE,bauF,bfmR, bfmS,csuA,csuA/B,csuB,csuC,csuD,csuE,entE,ompA,pgaA,pga B,pgaC,pgaD,plc,plcD

abaI,abaR,adeF,adeG,adeH,bap,barA,barB,basA,basB,basC,bas D,basF,basG,basH,basI,basJ,bauB,bauC,bauD,bauE,bauF,bfmR, bfmS,csuA,csuA/B,csuB,csuC,csuD,csuE,entE,ompA,pgaA,pga B,pgaC,pgaD,plc,plcD

abaI,abaR,adeF,adeG,adeH,bap,barA,barB,basA,basB,basC,bas D,basF,basG,basH,basI,basJ,bauB,bauC,bauD,bauE,bauF,bfmR, bfmS,csuA,csuA/B,csuB,csuC,csuD,csuE,entE,ompA,pgaA,pga B,pgaC,pgaD,plc,plcD

Different alleles

106

108

111

139

142

143

144

**TABLE 7 (Continued)**

Isolate

74SM01

96SM

61SM01

MGTN

103SM

MONUR

5MO

TG22150

Accession number

FUES01

FUEP01

FUEL01

FUEO01

FUEM01

FUET01

FUEY01

ASFO01

ST

944.0

944.0

944.0

944.0

944.0

944.0

944.0

944.0

Host

–

–

–

–

–

–

–

Human

Disease

–

–

–

–

–

–

–

–

Isolation Source

–

–

–

–

–

–

–

Tracheal aspirate

Country State

–

–

–

–

–

–

–

–

Collection Year

–

–

–

–

–

–

–

–

Antimicrobial resistance gene

aac(6′)-Ian,ant(2″)-Ia,aph(3′)- Ia,blaADC-25,blaOXA- 90,floR,sul2

aac(6′)-Ian,ant(2″)-Ia,aph(3′)- Ia,blaADC-25,blaOXA- 90,floR,sul2

aac(6′)-Ian,ant(2″)-Ia,aph(3′)- Ia,blaADC-25,blaOXA- 90,floR,sul2

aac(6′)-Ian,ant(2″)-Ia,aph(3′)- Ia,blaADC-25,blaOXA- 90,floR,sul2

ant(2″)-Ia,aph(3′)-Ia,blaADC- 25,blaOXA-90

aac(6′)-Ian,ant(2″)-Ia,aph(3′)- Ia,blaADC-25,blaOXA- 90,floR,sul2

aac(6′)-Ian,ant(2″)-Ia,aph(3′)- Ia,blaADC-25,blaOXA- 23,blaOXA-90,floR,sul2

aac(6′)-Ian,ant(2″)-Ia,aph(3′)- Ia,blaADC-25,blaCARB- 14,blaOXA-200

Virulence gene

abaI,abaR,adeF,adeG,adeH,bap,barA,barB,basA,basB,basC,bas D,basF,basG,basH,basI,basJ,bauB,bauC,bauD,bauE,bauF,bfmR, bfmS,csuA,csuA/B,csuB,csuC,csuD,csuE,entE,ompA,pgaA,pga B,pgaC,pgaD,plc,plcD

abaI,abaR,adeF,adeG,adeH,bap,barA,barB,basA,basB,basC,bas D,basF,basG,basH,basI,basJ,bauB,bauC,bauD,bauE,bauF,bfmR, bfmS,csuA,csuA/B,csuB,csuC,csuD,csuE,entE,ompA,pgaA,pga B,pgaC,pgaD,plc,plcD

abaI,abaR,adeF,adeG,adeH,bap,barA,barB,basA,basB,basC,bas D,basF,basG,basH,basI,basJ,bauB,bauC,bauD,bauE,bauF,bfmR, bfmS,csuA,csuA/B,csuB,csuC,csuD,csuE,entE,ompA,pgaA,pga B,pgaC,pgaD,plc,plcD

abaI,abaR,adeF,adeG,adeH,bap,barA,barB,basA,basB,basC,bas D,basF,basG,basH,basI,basJ,bauB,bauC,bauD,bauE,bauF,bfmR, bfmS,csuA,csuA/B,csuB,csuC,csuD,csuE,entE,ompA,pgaA,pga B,pgaC,pgaD,plc,plcD

abaI,abaR,adeF,adeG,adeH,bap,barA,barB,basA,basB,basC,bas D,basF,basG,basH,basI,basJ,bauB,bauC,bauD,bauE,bauF,bfmR, bfmS,csuA,csuA/B,csuB,csuC,csuD,csuE,entE,ompA,pgaA,pga B,pgaC,pgaD,plc,plcD

abaI,abaR,adeF,adeG,adeH,bap,barA,barB,basA,basB,basC,bas D,basF,basG,basH,basI,basJ,bauB,bauC,bauD,bauE,bauF,bfmR, bfmS,csuA,csuA/B,csuB,csuC,csuD,csuE,entE,ompA,pgaA,pga B,pgaC,pgaD,plc,plcD

abaI,abaR,adeF,adeG,adeH,bap,barA,barB,basA,basB,basC,bas D,basF,basG,basH,basI,basJ,bauB,bauC,bauD,bauE,bauF,bfmR, bfmS,csuA,csuA/B,csuB,csuC,csuD,csuE,entE,ompA,pgaA,pga B,pgaC,pgaD,plc,plcD

abaI,abaR,adeF,adeG,adeH,bap,barA,barB,basA,basB,basC,bas D,basF,basG,basH,basI,basJ,bauB,bauC,bauD,bauE,bauF,bfmR, bfmS,csuA,csuA/B,csuB,csuC,csuD,csuE,entE,ompA,pgaA,pga B,pgaC,pgaD,plc,plcD

Different alleles

147

149

150

150

152

152

157

158

**TABLE 7 (Continued)**

Isolate

25C30

TG28341

TG22142

TG41016

TG31301

TG40982

TG41245

TG22146

Accession number

FUER01

RFCW01

ASFL01

RFDJ01

RFDB01

RFBM01

RFBF01

ASFM01

ST

944.0

944.0

944.0

944.0

944.0

944.0

944.0

944.0

Host

–

Human

Human

Human

Human

Human

Human

Human

Disease

–

–

–

–

–

–

–

–

Isolation Source

–

Trach asp

Tracheal aspirate

Trach asp

Trach asp

Trach asp

Urine

Wound

Country State

–

USA:

Arizona

–

USA:

Arizona

USA:

Arizona

USA:

Arizona

USA:

Arizona

-

Collection Year

–

2012-02-23

–

2012-07-06

2012-03-06

2012-07-06

2012-07-06

-

Antimicrobial resistance gene

aac(6′)-Ian,ant(2″)-Ia,aph(3′)- Ia,blaADC-25,blaOXA- 545,floR,sul2

aac(6′)-Ian,ant(2″)-Ia,aph(3′)- Ia,blaADC-25,blaCARB- 14,blaOXA-200,floR,sul2

aac(6′)-Ian,ant(2″)-Ia,aph(3′)- Ia,blaADC-25,blaCARB- 14,blaOXA-200,floR,sul2

blaADC-25,blaOXA-200

aac(6′)-Ian,ant(2″)-Ia,aph(3′)- Ia,blaADC-25,blaCARB- 14,blaOXA-200,floR,sul2

aac(6′)-Ian,ant(2″)-Ia,aph(3′)- Ia,blaADC-25,blaCARB- 14,blaOXA-200,floR,sul2

aac(6′)-Ian,ant(2″)-Ia,aph(3′)- Ia,blaADC-25,blaCARB- 14,blaOXA-200,floR,sul2

aac(6′)-Ian,ant(2″)-Ia,aph(3′)- Ia,blaADC-25,blaCARB- 14,blaOXA-200,floR,sul2

Virulence gene

abaI,abaR,adeF,adeG,adeH,bap,barA,barB,basA,basB,basC,bas D,basF,basG,basH,basI,basJ,bauB,bauC,bauD,bauE,bauF,bfmR, bfmS,csuA,csuA/B,csuB,csuC,csuD,csuE,entE,ompA,pgaA,pga B,pgaC,pgaD,plc,plcD

abaI,abaR,adeF,adeG,adeH,bap,barA,barB,basA,basB,basC,bas D,basF,basG,basH,basI,basJ,bauB,bauC,bauD,bauE,bauF,bfmR, bfmS,csuA,csuA/B,csuB,csuC,csuD,csuE,entE,ompA,pgaA,pga B,pgaC,pgaD,plc,plcD

abaI,abaR,adeF,adeG,adeH,bap,barA,barB,basA,basB,basC,bas D,basF,basG,basH,basI,basJ,bauB,bauC,bauD,bauE,bauF,bfmR, bfmS,csuA,csuA/B,csuB,csuC,csuD,csuE,entE,ompA,pgaA,pga B,pgaC,pgaD,plc,plcD

abaI,abaR,adeF,adeG,adeH,bap,barA,barB,basA,basB,basC,bas D,basF,basG,basH,basI,basJ,bauB,bauC,bauD,bauE,bauF,bfmR, bfmS,csuA,csuA/B,csuB,csuC,csuD,csuE,ompA,pgaA,pgaB,pga C,pgaD,plc,plcD

abaI,abaR,adeF,adeG,adeH,bap,barA,barB,basA,basB,basC,bas D,basF,basG,basH,basI,basJ,bauB,bauC,bauD,bauE,bauF,bfmR, bfmS,csuA,csuA/B,csuB,csuC,csuD,csuE,entE,ompA,pgaA,pga B,pgaC,pgaD,plc,plcD

abaI,abaR,adeF,adeG,adeH,bap,barA,barB,basA,basB,basC,bas D,basF,basG,basH,basI,basJ,bauB,bauC,bauD,bauE,bauF,bfmR, bfmS,csuA,csuA/B,csuB,csuC,csuD,csuE,entE,ompA,pgaA,pga B,pgaC,pgaD,plc,plcD

abaI,abaR,adeF,adeG,adeH,bap,barA,barB,basA,basB,basC,bas D,basF,basG,basH,basI,basJ,bauB,bauC,bauD,bauE,bauF,bfmR, bfmS,csuA,csuA/B,csuB,csuC,csuD,csuE,ompA,pgaA,pgaB,pga C,pgaD,plc,plcD

abaI,abaR,adeF,adeG,adeH,bap,barA,barB,basA,basB,basC,bas D,basF,basG,basH,basI,basJ,bauB,bauC,bauD,bauE,bauF,bfmR, bfmS,csuA,csuA/B,csuB,csuC,csuD,csuE,entE,ompA,pgaA,pga B,pgaC,pgaD,plc,plcD

Different alleles

159

159

160

160

161

161

163

164

**TABLE 7 (Continued)**

Isolate

TG29428

TG41883

14,336

2RED09

20C15

abaum007

PR355

PR308

Accession number

RFEC01

RFDO01

FUEN01

FUEU01

FUEQ01

UWXI01

NGCI01

NGBY01

ST

944.0

944.0

944.0

944.0

944.0

944.0

944.0

944.0

Host

Human

Human

–

–

–

Human

–

–

Disease

–

–

–

–

–

–

–

–

Isolation Source

Trach asp

Trach asp

–

–

–

Hospital

–

–

Country State

USA:

Arizona

USA:

Arizona

–

–

–

Switzerland

–

–

Collection Year

2012-03-06

2012-07-06

–

–

–

2010

–

–

Antimicrobial resistance gene

aac(6′)-Ian,ant(2″)-Ia,aph(3′)- Ia,blaADC-25,blaCARB- 14,blaOXA-200,floR,sul2

aac(6′)-Ian,ant(2″)-Ia,aph(3′)- Ia,blaADC-25,blaCARB- 14,blaOXA-200,floR,sul2

ant(2″)-Ia,aph(3′)-Ia,blaADC- 25,blaOXA-58,blaOXA-90

aac(6′)-Ian,ant(2″)-Ia,aph(3′)- Ia,blaADC-25,blaOXA- 58,blaOXA-90,floR,sul2

aac(6′)-Ian,ant(2″)-Ia,aph(3′)- Ia,blaADC-25,blaOXA- 23,blaOXA-58,blaOXA- 90,floR,sul2

aac(6′)-Ian,ant(2″)-Ia,aph(3′)- Ia,blaADC-25,blaCARB- 14,blaOXA-90,sul2

blaADC-25,blaOXA-90

blaADC-25,blaOXA-90

Virulence gene

abaI,abaR,adeF,adeG,adeH,bap,barA,barB,basA,basB,basC,bas D,basF,basG,basH,basI,basJ,bauB,bauC,bauD,bauE,bauF,bfmR, bfmS,csuA,csuA/B,csuB,csuC,csuD,csuE,ompA,pgaA,pgaB,pga C,pgaD,plc,plcD

abaI,abaR,adeF,adeG,adeH,bap,barA,barB,basA,basB,basC,bas D,basF,basG,basH,basI,basJ,bauB,bauC,bauD,bauE,bauF,bfmR, bfmS,csuA,csuA/B,csuB,csuC,csuD,csuE,ompA,pgaA,pgaB,pga C,pgaD,plc,plcD

abaI,abaR,adeF,adeG,adeH,bap,barA,barB,basA,basB,basC,bas D,basF,basG,basH,basI,basJ,bauB,bauC,bauD,bauE,bauF,bfmR, bfmS,csuA,csuA/B,csuB,csuC,csuD,csuE,entE,ompA,pgaA,pga B,pgaC,pgaD,plc,plcD

abaI,abaR,adeF,adeG,adeH,bap,barA,barB,basA,basB,basC,bas D,basF,basG,basH,basI,basJ,bauB,bauC,bauD,bauE,bauF,bfmR, bfmS,csuA,csuA/B,csuB,csuC,csuD,csuE,entE,ompA,pgaA,pga B,pgaC,pgaD,plc,plcD

abaI,abaR,adeF,adeG,adeH,bap,barA,barB,basA,basB,basC,bas D,basF,basG,basH,basI,basJ,bauB,bauC,bauD,bauE,bauF,bfmR, bfmS,csuA,csuA/B,csuB,csuC,csuD,csuE,entE,ompA,pgaA,pga B,pgaC,pgaD,plc,plcD

abaI,abaR,adeF,adeG,adeH,bap,barA,barB,basA,basB,basC,bas D,basF,basG,basH,basI,basJ,bauB,bauC,bauD,bauE,bauF,bfmR, bfmS,csuA,csuA/B,csuB,csuC,csuD,csuE,entE,ompA,pgaA,pga B,pgaC,pgaD,plc,plcD

abaI,abaR,adeF,adeG,adeH,bap,barA,barB,basA,basB,basC,bas D,basF,basG,basH,basI,basJ,bauB,bauC,bauD,bauE,bauF,bfmR, bfmS,csuA,csuA/B,csuB,csuC,csuD,csuE,entE,ompA,pgaA,pga B,pgaC,pgaD,plc,plcD

abaI,abaR,adeF,adeG,adeH,bap,barA,barB,basA,basB,basC,bas D,basF,basG,basH,basI,basJ,bauB,bauC,bauD,bauE,bauF,bfmR, bfmS,csuA,csuA/B,csuB,csuC,csuD,csuE,entE,ompA,pgaA,pga B,pgaC,pgaD,plc,plcD

Different alleles

164

166

175

184

189

203

211

212

*(Continued)*

**TABLE 7 (Continued)**

Isolate

UH5207

855,125

1,096,934

ABBL025

MRSN7735

PR371

PR385

ABUH393

3,909

Accession number

AYFP01

JMNT01

JEXM01

LLDF01

VHDV01

NGCY01

NGES01

MSMS01

AEOZ01

ST

944.0

944.0

944.0

944.0

944.0

944.0

944.0

Host

Human

Human

Human

Human

Human

-

-

Human

Human

Disease

-

-

-

Bacteremia

-

-

-

-

Pneumonia

Isolation Source

Catheter

Perirectal

Perirectal

Blood

Wound

-

-

Urine

Blood

Country State

USA:

Cleveland, OH

USA:

Maryland

USA:

Maryland

USA:

Chicago

USA

-

-

USA: Ohio

-

Collection Year

10/12/07

2012

2012

24-Apr-2006

2006

-

-

05-Aug-2009

2006

Antimicrobial resistance gene

blaADC-25,blaOXA-90

blaADC-25,blaOXA-90

blaADC-25,blaOXA-90

blaADC-25,blaOXA-90

blaADC-25,blaOXA-90

blaADC-25,blaOXA-90

blaADC-25,blaOXA-90

blaADC-25,blaOXA- 23,blaOXA-90

ant(2″)-Ia,aph(3′)-Ia,blaADC- 25,blaOXA-58,blaOXA- 90,floR,sul2

Virulence gene

abaI,abaR,adeF,adeG,adeH,bap,barA,barB,basA,basB,basC,bas D,basF,basG,basH,basI,basJ,bauB,bauC,bauD,bauE,bauF,bfmR, bfmS,csuA,csuA/B,csuB,csuC,csuD,csuE,entE,ompA,pgaA,pga B,pgaC,pgaD,plc,plcD

abaI,abaR,adeF,adeG,adeH,bap,barA,barB,basA,basB,basC,bas D,basF,basG,basH,basI,basJ,bauB,bauC,bauD,bauE,bauF,bfmR, bfmS,csuA,csuA/B,csuB,csuC,csuD,csuE,entE,ompA,pgaA,pga B,pgaC,pgaD,plc,plcD

abaI,abaR,adeF,adeG,adeH,bap,barA,barB,basA,basB,basC,bas D,basF,basG,basH,basI,basJ,bauB,bauC,bauD,bauE,bauF,bfmR, bfmS,csuA,csuA/B,csuB,csuC,csuD,csuE,entE,ompA,pgaA,pga B,pgaC,pgaD,plc,plcD

abaI,abaR,adeF,adeG,adeH,bap,barA,barB,basA,basB,basC,bas D,basF,basG,basH,basI,basJ,bauB,bauC,bauD,bauE,bauF,bfmR, bfmS,csuA,csuA/B,csuB,csuC,csuD,csuE,entE,ompA,pgaA,pga B,pgaC,pgaD,plc,plcD

abaI,abaR,adeF,adeG,adeH,bap,barA,barB,basA,basB,basC,bas D,basF,basG,basH,basI,basJ,bauB,bauC,bauD,bauE,bauF,bfmR, bfmS,csuA,csuA/B,csuB,csuC,csuD,csuE,entE,ompA,pgaA,pga B,pgaC,pgaD,plc,plcD

abaI,abaR,adeF,adeG,adeH,bap,barA,barB,basA,basB,basC,bas D,basF,basG,basH,basI,basJ,bauB,bauC,bauD,bauE,bauF,bfmR, bfmS,csuA,csuA/B,csuB,csuC,csuD,csuE,entE,ompA,pgaA,pga B,pgaC,pgaD,plc,plcD

abaI,abaR,adeF,adeG,adeH,bap,barA,barB,basA,basB,basC,bas D,basF,basG,basH,basI,basJ,bauB,bauC,bauD,bauE,bauF,bfmR, bfmS,csuA,csuA/B,csuB,csuC,csuD,csuE,entE,ompA,pgaA,pga B,pgaC,pgaD,plc,plcD

abaI,abaR,adeF,adeG,adeH,bap,barA,barB,basA,basB,basC,bas D,basF,basG,basH,basI,basJ,bauB,bauC,bauD,bauE,bauF,bfmR, bfmS,csuA,csuB,csuC,csuD,csuE,entE,ompA,pgaA,pgaB,pgaC, pgaD,plc,plcD

abaI,abaR,adeG,adeH,bap,barA,barB,basA,basB,basC,basD,bas F,basG,basH,basI,basJ,bauB,bauC,bauD,bauE,bauF,bfmR,bfmS, csuA,csuA/B,csuB,csuC,csuD,csuE,entE,pgaA,pgaB,plc,plcD

Different alleles

212

223

232

234

240

254

254

266

298

*(Continued)*

1965

1966

1967

1968

1969

1970

1971

1972

1973

1974

1975

1976

1977

1978

1979

1980

1981

1982

1983

1984

1985

1986

1987

1988

1989

1990

1991

1992

1993

1994

1995

1996

1997

1998

1999

2000

2001

2002

2003

2004

2005

2006

2007

2008

2009

2010

2011

2012

2013

2014

2015

2016

2017

2018

2019

2020

2021

2022

**TABLE 7 (Continued)**

Isolate

ABBL026

abaum001

831,240

830

3,365

4,022

Accession number

LLDG01

UWVY01

JEYO01

WIVL01

WIVN01

WIVO01

ST

944.0

944.0

1104.0

944.0

944.0

Host

Human

Human

Human

Human

Human

Human

Disease

Bacteremia

-

-

Staph infection

Staph infection

Staph infection

Isolation Source

Blood

Hospital

Perirectal

Western Georgia

Western Georgia

Western Georgia

Country State

USA:

Chicago

Switzerland

USA:

Maryland

Georgia: Tbilisi

Georgia: Tbilisi

Georgia: Tbilisi

Collection Year

01-May-2006

2017

2012

2018

2018

2018

Antimicrobial resistance gene

blaADC-25,blaOXA-90

aac(6′)-Ian,ant(2″)-Ia,aph(3′)- Ia,blaADC-25,blaOXA- 545,floR,sul2

blaADC-25,blaOXA-90

aadA5,armA,blaADC- 25,blaCTX-M-124,blaOXA-

90,mph(E),msr(E),sul1

aac(6′)-Ian,armA,blaADC- 25,blaCARB-14,blaCTX-M-

124,blaOXA-

90,floR,mph(E),msr(E),sul1

aadA5,armA,blaADC- 25,blaOXA- 90,mph(E),msr(E),sul1

Virulence gene

abaI,abaR,adeF,adeG,adeH,bap,barA,barB,basA,basB,basC,bas D,basF,basG,basH,basI,basJ,bauB,bauC,bauD,bauE,bauF,bfmR, bfmS,csuA,csuA/B,csuB,csuC,csuD,csuE,entE,ompA,pgaA,pga B,pgaC,pgaD,plc,plcD

abaI,abaR,adeF,adeG,adeH,bap,barA,barB,basA,basB,basC,bas D,basF,basG,basH,basI,basJ,bauB,bauC,bauD,bauE,bauF,bfmR, bfmS,csuA,csuA/B,csuB,csuC,csuD,csuE,entE,ompA,pgaA,pga B,pgaC,pgaD,plc,plcD

abaI,abaR,adeF,adeG,adeH,bap,barA,barB,basA,basB,basC,bas D,basF,basG,basH,basI,basJ,bauB,bauC,bauD,bauE,bauF,bfmR, bfmS,csuA,csuA/B,csuB,csuC,csuD,csuE,entE,ompA,pgaA,pga B,pgaC,pgaD,plc,plcD

adeF,adeG,adeH,bap,barA,barB,basA,basB,basC,basD,basF,bas G,basH,basI,basJ,bauB,bauC,bauD,bauE,bauF,bfmR,bfmS,csuA

,csuA/B,csuB,csuC,csuD,csuE,entE,ompA,pgaA,pgaB,pgaC,pga D,plc,plcD

abaI,abaR,adeF,adeG,adeH,bap,barA,barB,basA,basB,basC,bas D,basF,basG,basH,basI,basJ,bauB,bauC,bauD,bauE,bauF,bfmR, bfmS,csuA,csuA/B,csuB,csuC,csuD,csuE,entE,ompA,pgaA,pga B,pgaC,pgaD,plc,plcD

abaI,abaR,adeF,adeG,adeH,bap,barA,barB,basA,basB,basC,bas D,basF,basG,basH,basI,basJ,bauB,bauC,bauD,bauE,bauF,bfmR, bfmS,csuA,csuA/B,csuB,csuC,csuD,csuE,entE,ompA,pgaA,pga B,pgaC,pgaD,plc,plcD

Different alleles

333

343

423

54

81

84

2081

2082

2083

2084

2085

2086

2087

2088

2089

2090

2091

2092

2093

2094

2095

2096

2097

2098

2099

2100

2101

2102

2103

2104

2105

2106

2107

2108

2109

2110

2111

2112

2113

2114

2115

2116

2117

2118

2119

2120

2121

2122

2123

2124

2125

2126

2127

2128

2129

2130

2131

2132

2133

2134

2135

2136

2137

2138
